# Genome-wide association study for resistance to *Pseudomonas syringae* pv. *garcae* in *Coffea arabica*

**DOI:** 10.3389/fpls.2022.989847

**Published:** 2022-10-18

**Authors:** Caroline Ariyoshi, Gustavo César Sant’ana, Mariane Silva Felicio, Gustavo Hiroshi Sera, Livia Maria Nogueira, Lucas Mateus Rivero Rodrigues, Rafaelle Vecchia Ferreira, Bruna Silvestre Rodrigues da Silva, Mário Lúcio Vilela de Resende, Suzete Aparecida Lanza Destéfano, Douglas Silva Domingues, Luiz Filipe Protasio Pereira

**Affiliations:** ^1^ Programa de pós-graduação em Genética e Biologia Molecular, Universidade Estadual de Londrina (UEL), Centro de Ciâncias Biológicas, Londrina, Brazil; ^2^ Área de Melhoramento Genético e Propagação Vegetal, Instituto de Desenvolvimento Rural do Paraná (IDR-Paraná), Londrina, Brazil; ^3^ Tropical Melhoramento & Genética (TMG), Londrina, Brazil; ^4^ Programa de pós-graduação em Ciências Biológicas (Genética), Universidade Estadual Paulista “Júlio de Mesquita Filho“ (UNESP), Instituto de Biociências, Campus de Botucatu, Botucatu, Brazil; ^5^ Centro de Café Alcides Carvalho, Instituto Agronômico (IAC), Campinas, Brazil; ^6^ Departamento de Fitopatologia, Universidade Federal de Lavras (UFLA), Lavras, Brazil; ^7^ Instituto Biológico (IB), Campinas, Brazil; ^8^ Departamento de Genética, Escola Superior de Agricultura Luiz de Queiroz, Universidade de São Paulo (USP), Piracicaba, Brazil; ^9^ Empresa Brasileira de Pesquisa Agropecuária (EMBRAPA-Café), Brasília, Brazil

**Keywords:** coffee domestication, bacterial halo blight, plant disease, single-locus genome-wide association, multi-locus genome-wide association

## Abstract

Bacteria halo blight (BHB), a coffee plant disease caused by *Pseudomonas syringae* pv. *garcae*, has been gaining importance in producing mountain regions and mild temperatures areas as well as in coffee nurseries. Most *Coffea arabica* cultivars are susceptible to this disease. In contrast, a great source of genetic diversity and resistance to BHB are found in *C. arabica* Ethiopian accessions. Aiming to identify quantitative trait nucleotides (QTNs) associated with resistance to BHB and the influence of these genomic regions during the domestication of *C. arabica*, we conducted an analysis of population structure and a Genome-Wide Association Study (GWAS). For this, we used genotyping by sequencing (GBS) and phenotyping for resistance to BHB of a panel with 120 C*. arabica* Ethiopian accessions from a historical FAO collection, 11 C*. arabica* cultivars, and the BA-10 genotype. Population structure analysis based on single-nucleotide polymorphisms (SNPs) markers showed that the 132 accessions are divided into 3 clusters: most wild Ethiopian accessions, domesticated Ethiopian accessions, and cultivars. GWAS, using the single-locus model MLM and the multi-locus models mrMLM, FASTmrMLM, FASTmrEMMA, and ISIS EM-BLASSO, identified 11 QTNs associated with resistance to BHB. Among these QTNs, the four with the highest values of association for resistance to BHB are linked to *g000* (Chr_0_434_435) and *g010741* genes, which are predicted to encode a serine/threonine-kinase protein and a nucleotide binding site leucine-rich repeat (NBS-LRR), respectively. These genes displayed a similar transcriptional downregulation profile in a *C. arabica* susceptible cultivar and in a *C. arabica* cultivar with quantitative resistance, when infected with *P. syringae* pv. *garcae*. However, peaks of upregulation were observed in a *C. arabica* cultivar with qualitative resistance, for both genes. Our results provide SNPs that have potential for application in Marker Assisted Selection (MAS) and expand our understanding about the complex genetic control of the resistance to BHB in *C. arabica*. In addition, the findings contribute to increasing understanding of the *C. arabica* domestication history.

## Introduction

Coffee (*Coffea* sp.) is one of the most important crops in the world, especially for tropical countries. Although the genus *Coffea* contains 124 species, only two have economic importance, *C. arabica* L. and *C. canephora* Pierre, corresponding to 59.96 and 40.04% of the world production, respectively (Davis et al., 2011; International Coffee Organization, 2020). *C. arabica* is an autogamous and tetraploid species (2n = 4x = 44) with two diploid ancestors: *C. canephora* and *C. eugenioides* (Lashermes et al., 1999; Clarindo and Carvalho, 2008). It was originated in South-West Ethiopia and South Sudan region and traditionally is cultivated at high altitudes. On this conditions, *C. arabica* is known to provide better beverage quality (Bertrand et al., 2006; Davis et al., 2012; Worku et al., 2018).

Disease incidence is one of the main problems in the cultivation of *C. arabica*, affecting both production and quality. Among the bacterial diseases, we highlight the bacterial halo blight (BHB) caused by *Pseudomonas syringae* pv. *garcae* (Amaral et al., 1956). This disease is prevalent at high altitudes, due to cold winds (Maciel et al., 2018). Worldwide, there are reports of its occurrence in Kenya, Ethiopia, China, and Brazil (Ramos and Shavdia, 1976; Adugna et al., 2012; Xuehui et al., 2013; Rodrigues et al., 2017a). BHB management, based on windbreaks, is not always possible on coffee farms and management based on chemicals is environmentally undesirable. Both solutions increase the production costs and, generally, are not highly efficient in field conditions. Thus, the use of cultivars resistant to BHB is a better alternative for coffee producers.

During the plant-pathogen interaction, once the pathogens overcome the mechanical defense barriers of the plants, resistant plants activate receptors that can recognize the pathogen, activating signaling pathways that drive the defense response gene expression. These defense genes can reduce pathogen infection and colonization through their antimicrobial and cell wall enforcement properties (Andersen et al., 2018). At the first level of recognition, pathogen conserved molecular patterns, known as pathogen-associated molecular patterns (PAMPs), can be detected by host proteins, known as pattern recognition receptors (PRRs), which are capable of activating PAMP-triggered immunity (PTI). The plasma membrane localized plant receptors, such as receptor-like kinases (RLKs) and receptor-like proteins (RLPs) are examples of PRR proteins (Macho and Zipfel, 2015). However, some specialized pathogens produce effectors that can inhibit the PTI (Boller and Felix, 2009).

The second level of recognition involves R proteins capable of detecting effectors and activates effector-triggered immunity (ETI) (Jones and Dangl, 2006; Yuan et al., 2021). Among the R proteins, the class with the greatest representativeness is the nucleotide binding sites leucine-rich repeats (NBSs-LRRs) (Coll et al., 2011). The NBS-LRR and effector interaction can occur directly or indirectly. In the indirect way, the NBS-LRR monitors the binding of the effector with a “decoy” protein (Cui et al., 2015). Both ETI and PTI can activate the hypersensitive response (HR) (Kushalappa et al., 2016). Furthermore, the ETI robustness depends on the PTI (Yuan et al., 2021). The HR is considered as qualitative resistance controlled by few genes that have major effect (R genes) (Jones et al., 2016), and the qualitative resistance is defined by two categories: resistant and susceptible plants (Corwin and Kliebenstein, 2017).

The third line of defense is the absence or weakening of PTI and/or ETI, without HR (Niks et al., 2015). In this type of resistance, some antimicrobial and cell wall enforcement products are unavailable or available in reduced amounts, which allows the advance of pathogens (Boyd et al., 2012). This type of resistance can occur in a constitutive or an induced way and correspond to quantitative resistance (Kushalappa and Gunnaiah, 2013). In the quantitative resistance, the individuals can be divided into a continuous distribution between the resistance and susceptibility, which is related to the number and amount of defense products available (Kushalappa and Gunnaiah, 2013; Corwin and Kliebenstein, 2017). The genes controlling quantitative resistance are located at multiple loci with variable effects (Pilet-Nayel et al., 2017).

Due to its reproductive biology and relatively recent origin, *C. arabica* has low genetic diversity (Lashermes et al., 2000). In addition, most *C. arabica* cultivars are derived from only two varieties Typica and Bourbon, a factor responsible for a genetic bottleneck during the cultivar development (Anthony et al., 2001). Consequently, several cultivars are susceptible to diseases and pests (van der Vossen et al., 2015).

In contrast to the lack of genetic diversity in *C. arabica* cultivars, greater diversity is found in *C. arabica* Ethiopian accessions. Due to this diversity, a survey organized by the Food and Agriculture Organization (FAO) between 1964 and 1965, collected these accessions in Ethiopia. The collection took place in two regions, the East and West of the Great Rift Valley (FAO: Food and Agriculture Organization, 1968). During the *C. arabica* domestication process, there was, probably, a movement of plants from the West to the East of the Great Rift Valley (Montagnon and Bouharmont, 1996; Scalabrin et al., 2020).

Several studies, based on this *C. arabica* Ethiopian germplasm, identified sources of desirable traits, such as resistance to BHB (Mohan et al., 1978); resistance to nematodes *Meloidogyne paranaensis*, *M. incognita*, and *M. exigua* (Anzueto et al., 2001; Fatobene et al., 2017; Holderbaum et al., 2020); resistance to coffee berry disease (van der Vossen and Walyaro, 2009); drought tolerance (Queiroz-Voltan et al., 2014); biochemical compounds which influence beverage quality (Bertrand et al., 2006); low caffeine content (Silvarolla et al., 2004); and other chemical compounds (Scholz et al., 2016).

A large number of genetic recombination historical events present among unrelated individuals allows high-resolution mapping through Genome-Wide Association Studies (GWAS) (Migicovsky and Myles, 2017). Recent association maps, using *C. arabica* Ethiopian accessions, identified alleles associated with the chemical composition of green grains (chlorogenic acids levels, caffeine, total sugars, sucrose, lipids, proteins, and tannins) (Sant’ana et al., 2018).

In this work, to explore the genetic diversity of *C. arabica* Ethiopian accessions as well as of selected cultivars, we perform a GWAS for *C. arabica* and *P. syringae* pv. *garcae* interaction. We also identify and validate candidate genes involved in qualitative resistance to BHB by expression analysis and discuss whether the QTNs associated with resistance to BHB may be inserted in genomic regions that were relevant during the *C. arabica* domestication history.

## Materials and methods

### Plant materials

A total of 132 C*. arabica* genotypes were used, including 120 Ethiopian accessions from the FAO collection (FAO: Food and Agriculture Organization, 1968), the BA-10 genotype, and 11 cultivars: IPR 99, IPR 100, IPR 101, IPR 102, IPR 103, IPR 104, IPR 105, IPR 107, Catuaí Vermelho IAC 99, Bourbon Vermelho, and IAPAR 59 (**Supplementary Table 1**).

Phenotype data, for resistance to BHB, were accessed from two previous works conducted at the Instituto de Desenvolvimento Rural do Paraná (23° 22’ S, 51° 10’ W, 585 m asl) in Londrina, Paraná State, Brazil (Mohan et al., 1978; Ito et al., 2008). The phenotyping of *C. arabica* Ethiopian accessions, Bourbon Vermelho cultivar, and BA-10 genotype was carried out in the field with two replicates in plants that were approximately 1-year-old. The isolate number 586 of *P. syringae* pv. *garcae* was used and evaluations were carried out 17 and 35 days after inoculation (dai). The two evaluations obtained the same results (Mohan et al., 1978). The phenotyping of IPR 99, IPR 100, IPR 101, IPR 102, IPR 103, IPR 104, IPR 105, IPR 107, IAPAR 59, and Catuaí Vermelho IAC 99 cultivars were also evaluated in the field, with nine replications in plants approximately 10-months-old, with natural infections (Ito et al., 2008). In those studies, a scale with a score from 0 to 5 was used, where 0 corresponds to highly resistant plants, 1 to resistant, 2 to moderately resistant, 3 to moderately susceptible, 4 to susceptible, and 5 to highly susceptible (**Supplementary Figure 1**).

The current work used the GBS data from DNA samples of these 132 C*. arabica* accessions (Sant’ana et al., 2018). This single-end sequencing was performed using the restriction enzyme *PstI*, through the platform Illumina HiSeq2000 in partnership with the Genomic Diversity Facility LIMS, at Cornell University (Ithaca, NY - USA). This work used the GBS data (1,083,002 tags) aligned to the *C. arabica* Et39 reference genome (Salojärvi et al., 2021), performed by Felicio (2020) which allowed identification of 159,000 SNPs.

### SNP filtering and linkage disequilibrium analyses

The 159,000 SNPs were filtered using TASSEL software version 5.2.53 (Bradbury et al., 2007). The filtering was performed with the parameters of the minimum allele frequency greater than 0.05 (MAF> 0.05), call rate> 0.8, and removal of monomorphic and indel sites. The data were also imputed by LD kNNi software, which is based on the k-nearest neighbor method (Money et al., 2015). The SNPs obtained after this filter quality were used for the population structure study and for the GWAS.

For the linkage disequilibrium (LD) analysis, the panel of 159,000 SNPs was filtered through the software TASSEL, version 5.2.53. The filters used were: MAF> 0.05, call rate> 0.25, and removal of monomorphic and indel sites. The SNPs corresponding to scaffolds that did not have a defined position during the genome assembly (Chr zero) were also removed. The squared correlation coefficients (*r^2^*) were calculated on sliding windows with 50 adjacent SNPs, in TASSEL version 5.2.53, and used to evaluate the extent of LD decay. The value of LD distance (in bp) was evaluated by a non-linear regression, at *r^2 =^* 0.2, in R studio software (RStudio Team, 2020).

### Population structure study

A dissimilarity matrix was built and used for hierarchical analyses, with the complete linkage method and the Euclidean distance, by the R package cluster, and visualized graphically through the factoextra package (Maechler et al., 2019; RStudio Team, 2020; Kassambara and Mundt, 2020).

A Principal Component Analysis (PCA) was performed using TASSEL software, version 5.2.53 (Bradbury et al., 2007). The dispersion plot from the first and second Principal Components (PCs) was conducted using the R package cluster and visualized graphically using the package ggfortify (Horikoshi and Tang, 2016).

Population structure was also analyzed using the Bayesian method, implemented in STRUCTURE 2.3.4 (Pritchard et al., 2000). We predefined the number of genetic clusters K from 2 to 10 in the population. The program was set on 10,000 as burn-in iteration, followed by 10,000 Markov chain Monte Carlo (MCMC) replications with 20 runs per K. We used the ΔK criterion in Structure Harvester software to estimate the upper-most level of structure (Evanno et al., 2005; Earl and von Holdt, 2012).

### Gwas

The associative mappings were conducted using a single-locus model and four multi-locus models. The single-locus model used was the mixed linear model (MLM), through the software TASSEL 5.2.53 (Yu et al., 2006; Bradbury et al., 2007). For MLM, the association threshold established, to reduce false positive associations, was Bonferroni. For the multi-locus approach, we used four models within the R package mrMLM: “random-SNP-effect MLM (rMLM) and a multi-locus mrMLM” (mrMLM) (Wang et al., 2016), FASTmrMLM (Tamba and Zhang, 2018), “fast multi-locus random-SNP-effect EMMA” (FASTmrEMMA) (Wen et al., 2017), and “integrative sure independence screening EM-Bayesian LASSO” (ISIS EM-BLASSO) (Tamba et al., 2017).

These multi-locus models have two stages. In the first stage, a single-locus model and a modified Bonferroni association significance threshold are used, producing an intermediate result. In the second stage, for the multi-locus model implementation, it is necessary to reduce the data dimensionality. Thus, only the SNPs with the greatest potential for association selected in the first stage composed the data for the second stage. The critical values for SNPs to integrate data for the second stage were: p-value ≤ 0.01, 0.01, 0.005, and 0.01 for mrMLM, FASTmrMLM, FASTmrEMMA, and ISIS EM-BLASSO, respectively. In the second stage, the association threshold for all models was the LOD score = 3. The QTNs associated above LOD = 3 corresponded to the final result.

The single-locus model was adjusted using the first five PCs based on the dissimilarity matrix and kinship matrix (K matrix). To verify the best adjusted performance for the multi-locus models, these were corrected by K matrix, K matrix + the first five PCs based on the 11,290 SNPs and K matrix + Q matrix. The Q matrix was based on the highest level of population structure (K = 2) identified from software STRUCTURE. For the single-locus MLM model, the K matrix was calculated using TASSEL 5.2.53 software, and for the multi-locus models, using the mrMLM package.

From the HapMap genotyping file, the presence or absence of the QTNs identified in the GWAS was performed in the 132 C*. arabica* accessions, with its respective resistance to BHB phenotype score. With this identification, the coefficient of correlation between the QTNs associated with resistance to BHB was calculated. The coefficient of correlation between each QTN identified in GWAS and the resistance to BHB, in the 132 C*. arabica* accessions, was also calculated. The coefficient of correlation calculation and the graphical visualization were made using the R packages Hmisc and corrplot, respectively (Harrell and Dupont, 2020; Wei and Simko, 2021).

### Identification of candidate genes

To understand the molecular basis of the *C. arabica* defense to BHB, we investigated the genomic regions of associated QTNs for possible candidate genes. A search for candidate genes was performed using the *C. arabica* Et039 genome functional annotation (Salojärvi et al., 2021), according to the LD threshold, upstream and downstream to the associated QTNs positions. Functional annotation and genomic position of the *C. arabica* genes was retrieved and examined from the *C. arabica* Et039 genome functional annotation. Subsequently, proteins with biotic stress response function were literature mined from annotated information.

### Plant materials for RT-qPCR

Plants of 5-months-old of *C. arabica* cultivars Catuaí Vermelho IAC 99 (susceptible), IAPAR 59 (quantitative resistance), and IPR 102 (qualitative resistance) were submitted to infection with *P. syringae* pv. *garcae* through the method established by Rodrigues et al. (2017b). The inoculum of *P. syringae* pv. *garcae* grown on agar for 48h (28 °C), was diluted in saline (0.85% NaCl) and standardized by spectrophotometry (A600 = 0.25) containing 10^8^ UFC/ml. Two pairs of the first expanded leaves of seedlings were inoculated with punctures through needles, previously dipped in the inoculum, at two points on each side of the central rib.

Leaves were collected before the inoculation at 0 hours (h) and, 10 minutes (min), 30 min, 1 h, 3 h, and 6 h, time after inoculation (tai), for RNA extraction. The experimental design for RT-qPCR was conducted with 3 plants of each cultivar for each collection time.

Two inoculated leaves, in each plant, were preserved to verify the inoculum efficiency and the disease evaluation was performed at 21 (dai). The damage in the inoculated plants was estimated using a scale from 0 to 5, where 0 = resistant, absence of symptoms; 1 = moderately resistant, initial water-soaking, up-to 10% of leaf area inoculated with symptoms; 2 = susceptible, water-soaking up to approximately 25% of affected area; 3 = up to 50% of the area inoculated with symptoms; 4 = up to 75% of the affected inoculated area; 5 = more than 75% of the inoculated area necrotic from the disease (Rodrigues et al., 2017b).

### Expression profile of candidate genes by RT-qPCR

The RNA extraction was carried out following the protocol of Chang et al. (1993). The cDNAs were synthesized from 2.5 μg of total RNA using the reverse transcriptase enzyme from the SuperScript^®^ III First-Strand Synthesis SuperMix kit (Invitrogen), according to the manufacturer’s instructions, with a final volume of 20 μl.

Specific pairs of primers were designed for the cDNA corresponding to *g000* (Chr_0_434_435) and *g010741* genes using Primer Express software (Thermo Fisher Scientific) (**Supplementary Table 5**). Primer efficiencies were performed through the standard curve of cDNA dilutions: 1:2, 1:4, 1:8, 1:16, 1:32, and 1:64. Every measurement of dilutions was performed in triplicate. The corresponding qPCR efficiencies (E) were calculated for primer pairs with the 7500 Fast Real-Time PCR System (Applied Biosystems), according to the equation E = (10^-1/slope^ - 1) × 100 (Rutledge and Stewart, 2008).

The transcriptional profile was evaluated using qPCR (7500 Fast Real-Time PCR System, Applied Biosystems). The reaction volumes was 25 μL containing 12.5 μL of 2x SYBR^®^ Green/ROX qPCR Master Mix (Applied Biosystems), 0.5 μL of each primer (10 μM), 10.5 μL of treated DEPC water, and 1 μL of cDNA diluted to a concentration of 20 ng/μL.

The reactions were prepared in technical triplicates and the thermocycling parameters used were: 10 min at 95 °C, followed by 40 cycles of amplification of 95 °C for 30 seconds and 60 °C for 60 seconds. Dissociation curves were analyzed to verify the specificity of the primers in the amplification reaction.

The relative gene transcription was assessed using ΔΔCt = ΔCt (sample) - ΔCt (normalizer), where the *GAPDH* gene was used as housekeeping to normalize the data (Barsalobres-Cavallari et al., 2009). The calibrator used to compare the transcriptional activities pattern, between the times after inoculation, was the 0 h for each cultivar. The transcript data were subjected to statistical analysis of variance (ANOVA) and the Tukey test, both at the level of 5% significance, through R studio software (RStudio Team, 2020).

### Haplotype analysis

From the genomic region corresponding to the *g010741* gene (CC-NBS-LRR) of *C. arabica* Et039 and the respective homologous genomic regions of *C. arabica* Caturra and, *C. arabica* var. Geisha, a haplotype analysis was performed. For this, the information available in the genome databases of *C. arabica* Et039 (Salojärvi et al., 2021), *C. arabica* Caturra (NCBI BioProject PRJNA506972), and *C. arabica* var. Geisha (Phytozome genome ID 453), was used.

The genomic region corresponding to the *C. arabica* Et039 *g010741* gene (Chr2_sg_E, position 32,109,397 bp – 32,111,166 bp) was used to identify the homologous genomic regions in Caturra and Geisha, through the BLASTn tool. Multiple alignment between the nucleotide sequences of the *C. arabica* Et039 *g010741* gene and the homologous nucleotide sequences of Caturra, and Geisha was carried out to identify SNPs, using MUSCLE (Edgar, 2004). The respective protein sequences were also aligned to identify non-synonymous SNPs (nsSNPs). SNPs inserted into conserved domains were investigated. For this, a protein sequence of 1,280 aa corresponding to the *g010741* gene was retrieve from the *C. arabica* Et039 functional annotation and used to the identification of conserved domains and their respective genomic positions, through the InterPro (Blum et al., 2021).

Near to the *g010741* gene are the QTNs Chr_2_sg_E_32049720_G and Chr_2_sg_E_32049728_G associated with resistance to BHB. Thus, in addition to the haplotype analysis described above, the identification of standard alleles or alternative alleles for the QTNs Chr_2_sg_E_32049720_G and Chr_2_sg_E_32049728_G, in the three *C. arabica* genotypes, was made. From the QTNs Chr_2_sg_E_32049720_G and Chr_2_sg_E_32049728_G positions in the Et039 genome (reference genome used to align the GBS data in this work), the identification of standard alleles or alternative alleles was made. Using the nucleotide sequence (60 bp) surrounding this QTNs in Et039, a search for homology in Caturra and Geisha genotypes, was performed. The search for homology was performed using the BLASTn tool.

### Relationship between the loci involved in resistance to BHB and the *C. arabica* domestication process

The frequency of QTNs identified in the GWAS was determined in the clusters “wild”, “domesticated”, and “cultivar” identified in the Bayesian approach population structure at K = 3. For this, *C. arabica* Ethiopian accessions classified as admixed were disregarded. The frequency of genotypes resistant to the BHB, based in a scale with a score from 0 to 5, was also determined in the clusters

## Results

### SNP characteristics

After the quality filters and imputation, we selected 11,290 SNPs for population structure analysis and GWAS. From this total, 5,024 are distributed in the *C. canephora* subgenome, 5,011 in the *C. eugenioides* subgenome, and 1,255 in chromosome (Chr) zero. The Chr zero corresponds to scaffolds that did not have a defined position during the genome assembly. The SNPs/Megabase (Mb) frequency was 13.57 and 11.63 for the *C. canephora* and *C. eugenioides* subgenome, respectively. Among the chromosomes a large difference was observed in the SNP/Mb frequency. The Chr2 of the *C. canephora* subgenome showed the highest density of SNPs (17.81) and the Chr9 of the *C. eugenioides* subgenome the lowest (8.41) (**Supplementary Figure 2** and **Supplementary Table 2**).

### Population structure

The population used in this work is compose of 111 C*. arabica* Ethiopian accessions collected at the West of the Great Rift Valley, nine collected at the East of the Great Rift Valley, 11 C*. arabica* cultivars, and the BA-10 genotype.

**Figure 1:**
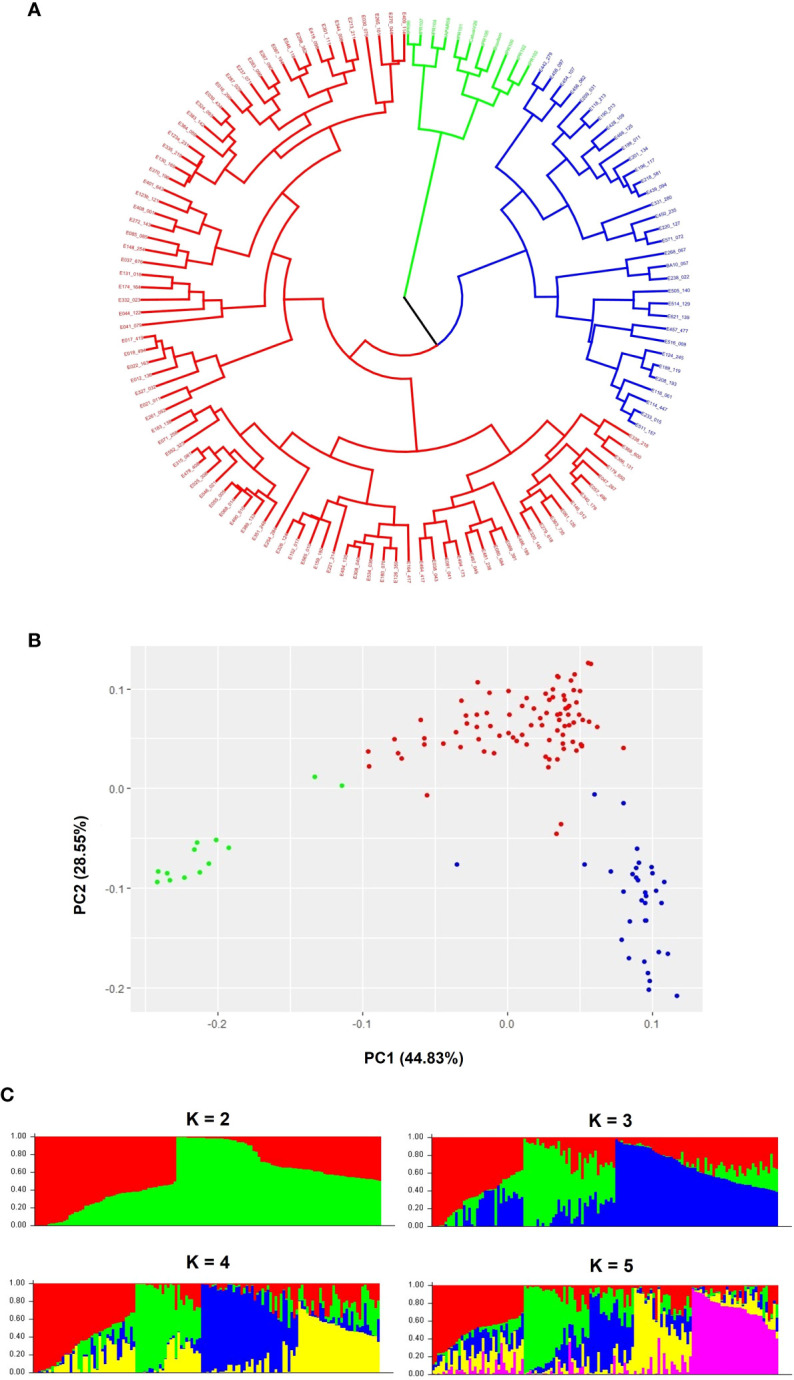
Genetic population analysis among 132 *Coffea arabica* genotypes using 11,290 SNPs. **(A)** Dendrogram; **(B)** principal coordinate analysis (PCA). Each point represents an accession, n = 132. In the PCA the accessions were colored according to the cluster: red “domesticated”, blue “wild”, and green “cultivar”. **(C)** Bar plots of the estimated membership coefficient of the 132 C*. arabica* accessions for K = 2, K = 3, K = 4 and, K = 5, using STRUCTURE.

The clustering based on the dissimilarity matrix of genetic distance of the 132 C*. arabica* accessions (**Supplementary Figure 4**), through the hierarchical approach, defined 3 clusters (**Figure 1A** and **Supplementary Table 1**). The cluster in blue is composed of 32 accessions, most of them collected in forests, which represents a lower domestication level, and the BA-10 genotype, which has a *C. liberica* introgression. The cluster in red is composed of 88 accessions collected, predominantly, in places with the description of naturalized, domesticated, farm open field and shadow. Naturalized, domesticated, and, mainly, farm open field represents a high domestication level. The cluster in green is composed of the *C. arabica* cultivars. Thus, the blue cluster was named “wild”, the red cluster “domesticated”, and the green cluster “cultivar”. Among the nine accessions collected at the East of the Great Rift Valley, one is in the “wild” cluster and eight are in the “domesticated” cluster.

The PCA scatter plot also showed three clusters, with accessions grouped according to the domestication level. The “wild” cluster in blue is composed of 33 accessions, predominantly, collected in forests, and the BA-10 genotype. The “domesticated” cluster in red is composed of 85 accessions, which were predominantly characterized as domesticated. However, different from the hierarchical analysis result, the “cultivar” cluster in green is composed of cultivars and more two *C. arabica* Ethiopian accessions collected in “farm open field”. Among the accessions collected at the East side of the Great Rift Valley, one is in the “wild” cluster, seven are in the “domesticated” cluster, and one is in the “cultivar” cluster (**Figure 1B** and **Supplementary Table 1**).

According to the Bayesian approach, using STRUCTURE software and Evanno’s criterion, the upper level of the population subdivision were K = 2. In addition, smaller peaks were observed at K = 3, and at K = 5 (**Supplementary Figure 5**), which might indicate another informative population structure. Therefore, the STRUCTURE results at K = 2, K =3, K = 4, and K = 5 were subject population genetics analyses. The population genetics analyses were based on the membership coefficient (≥ 0.6), so that the accessions could be assigned to a specific group. The membership coefficients of the *C. arabica* cultivars and C. *arabica* Ethiopian accessions at K =2, K = 3, K = 4, and K = 5 are available in the **Supplementary Material 1**.

At K = 2, the 11 cultivars and 30 C*. arabica* Ethiopian accessions, most of them characterized as domesticated, are grouped in the same cluster (in red). The second cluster (in green) is composed of the 32 most wild *C. arabica* Ethiopian accessions and 24 domesticated accessions. For this sub-populations structure, 34 C*. arabica* Ethiopian accessions and the BA-10 genotype (26,51%) were categorized as admixed. Among the accessions collected at the East of the Great Rift Valley, seven are in the red cluster, and two were classified as admixed (**Figure 1C** and **Supplementary Figure 6**).

A clearer separation of the domesticated *C. arabica* accessions from the cultivars, and of the domesticated *C. arabica* accessions from the most wild accessions could be observed at K = 3. The green cluster is composed of 11 cultivars and 5 domesticated accessions. The cluster in red is composed of 19 domesticated accessions and the BA-10 genotype. The blue cluster is composed of 33 accessions, predominantly, collected in forest. Thus, the green cluster corresponds to the “cultivar” cluster, the red to the “domesticated” cluster, and the blue to the “wild” cluster. At this population structure level, 63 (47,72%) accessions were categorized as admixed. Among the accessions collected at the East of the Great Rift Valley, one is in the “domesticated” cluster, three in the “cultivar” cluster, and five were classified as admixed (**Figure 1C** and **Supplementary Figure 6).** Although five accessions collected in the East were classified as admixed, it is possible to observe in three of these accessions a membership coefficient > 0.5 of the “cultivar” cluster. This result reinforces the genetic proximity between the cultivars and the accessions collected at the East of the Great Rift Valley.

The clustering pattern obtained at K = 4 revealed a “wild” cluster in blue with the BA-10 genotype and 28 accessions, predominantly, collected in forest, and a “cultivar” cluster in green with 11 cultivar and three domesticated accessions. However, at K = 4, were identified two “domesticated” clusters in red and in yellow, with 22 and 8 domesticated accessions, respectively. We did not identify any characteristic that could differentiate the accessions grouped in different “domesticated” clusters. For this four sub-populations, 59 (44,69%) accessions were classified as admixed. Among the accessions collected at the East of the Great Rift Valley, one is in the “cultivar” cluster, and eight were classified as admixed (**Figure 1C** and **Supplementary Figure 6**). In the same way as was observed at K = 3, three accessions collected at the East and classified as admixed have a membership coefficient > 0.5 of the “cultivar” cluster.

At K = 5, a “wild” cluster is represented in purple with 26 accessions, predominantly, collected in forest and the BA-10 genotype, and a “cultivar” cluster is represented in green with 11 cultivars and two domesticated accessions. Three “domesticated” clusters, in red, blue and yellow, with 11, 8, and 8 accessions, respectively, were identified. Here, we also did not identify characteristics that could distinguish the accessions grouped in the three different “domesticated” clusters. At K = 5, 63 (49,24%) accessions were classified as admixed. Among the accessions collected at the East of the Great Rift Valley, one is in the “domesticated” cluster in blue, and eight were classified as admixed (**Figure 1C** and **Supplementary Figure 6**). For this population structure level, only one accession collected in the East of the Great Rift Valley and classified as admixed has a membership coefficient > 0.5 of the “cultivar” cluster.

Although the higher delta K value was identified at K = 2, the population structure identified at K = 3 was suitable for grouping the *C. arabica* accessions according to their domestication level characteristics. This is in agreement with the hierarchical analysis and PCA results. In addition, the population structure level identified by the Bayesian approach at K =3, was suitable to show the most genetic similarity between accessions collected at the East of the Great Rift Valley and the cultivars. A large difference was observed in the number of accessions that compose the “domesticated” cluster, between the PCA analysis and the Bayesian approach. This is mainly because many accessions were classified as admixed in the Bayesian approach.

### Gwas

**Figure 2:**
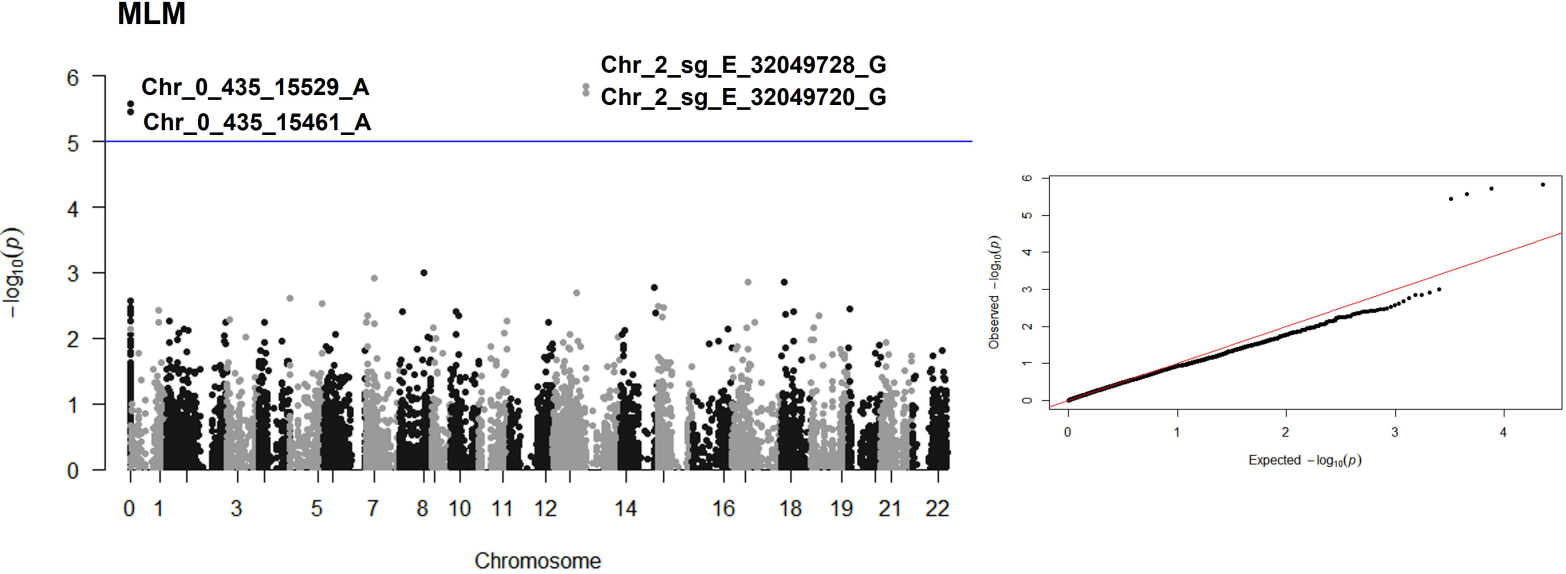
Manhattan plot and Q-Q plot corresponding to single-locus GWAS MLM for *C. arabica* and *P. syringae* pv. *garcae* interaction. The manhattan plot indicate the -log10(p-value) of SNP across the genome (y-axis) plotted against their respective position on each chromosome (x-axis). The blue line represents the Bonferroni corrected threshold. The chromosome (Chr) zero correspond to scaffolds that did not have a defined position during the genome assembly, Chr 1 to 11 correspond to canephora subgenome, Chr 12 to 22 correspond to eugenioides subgenome.

**Figure 3:**
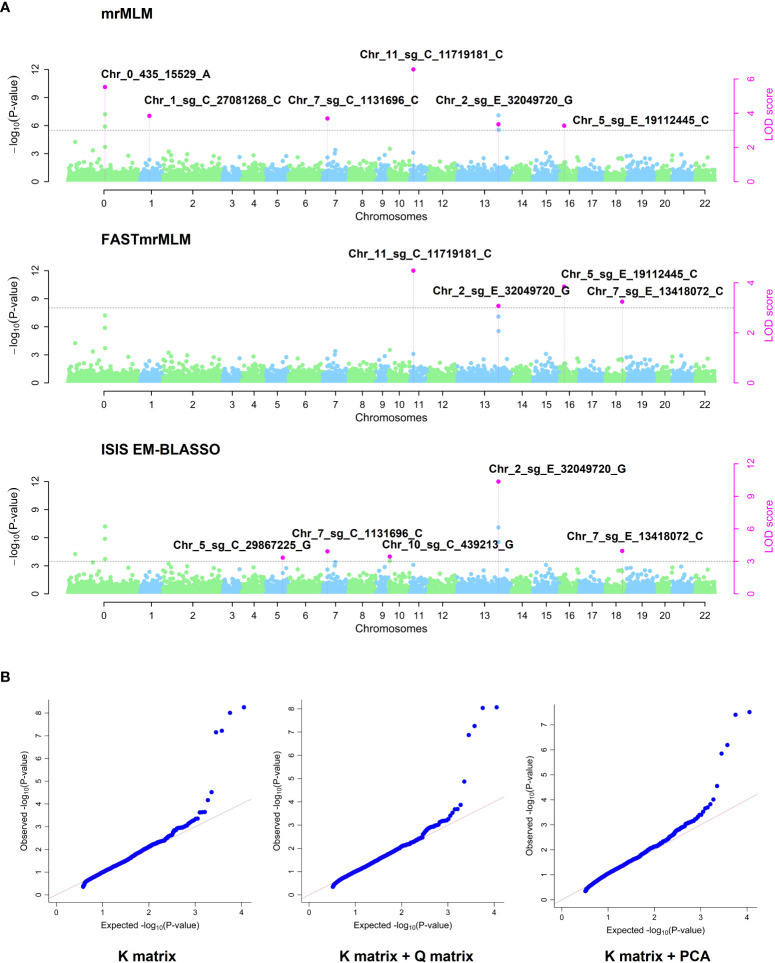
Multi-locus GWAS. **(A)** Manhattan plots corresponding to mrMLM, FASTmrMLM, and ISIS EM-BLASSO for C. *arabica* and *P. syringae* pv. *garcae* interaction. The manhattan plots indicate the -log10 (p-value) and the LOD score of SNP across the genome (y-axis) plotted against their respective position on each chromosome (x-axis). The chromosome (Chr) zero correspond to scaffolds that did not have a defined position during the genome assembly, Chr 1 to 11 correspond canephora subgenome, Chr 12 to 22 correspond to eugenioides subgenome. **(B)** Q-Q plots from multi-locus models adjusted by different population structure approaches. Kinship matrix (K matrix), principal component analysis (PCA), and the population structure K = 2 identified from software STRUCTURE (Q matrix).

From 11,290 SNPs obtained after quality filters, 11 QTNs associated with the *C. arabica* response to BHB, through the single-locus MLM model and the four multi-locus models, mrMLM, FASTmrMLM, FASTmrEMMA, and ISIS EM-BLASSO, were identified (**Table 1** and **Figures 2** and **3A**). The single-locus MLM model identified 4 QTNs. For the multi-locus GWAS approaches, a better fit of the data on Q-Q plot was observed in the model corrected with K matrix + PCA (**Figure 3B**). The multi-locus models mrMLM, FASTmrMLM, and ISIS EM-BLASSO, adjusted with K matrix + PCA, identified 6, 4, and 5 QTNs, respectively. The FASTmrEMMA did not identify associated QTNs.

**Table 1:** Description of QTNs associated with resistance to BHB in *C. arabica* through the MLM, mrMLM, FASTmrMLM, and ISIS EM-BLASSO models.

				MLM			mrMLM				FASTmrMLM				ISIS EM-BLASSO			
QTN^1^	Chr^2^	position (pb)^3^	allele standard/ alternative	-log10 (p-value)	QTN effect	*r^2^* (%)^4^	-log10 (p-value)	LOD SCORE	QTN effect	*r* ^2^(%)^4^	-log10 (p-value)	LOD SCORE	QTN effect	*r* ^2^(%)^4^	-log10 (p-value)	LOD SCORE	QTN effect	*r* ^2^(%)^4^
**Chr_0_435_15461_A**	**Sca_434HRSCAF=435**	**15461**	**G/A**	5.45	1.48	20.16												
**Chr_0_435_15529_A**	**Sca_434HRSCAF=435**	**15529**	**G/A**	5.58	1.35	20.16	6.34	5.52	0.86	13								
**Chr_1_sg_C_27081268_C**	**1 sg C**	**27081268**	**T/C**				4.59	3.84	-1.29	15.94								
**Chr_5_sg_C_29867225_G**	**5 sg C**	**29867225**	**A/G**												4.04	3.33	-0.74	7.48
**Chr_7_sg_C_1131696_C**	**7 sg C**	**1131696**	**T/C**				4.42	3.69	0.97	9.31					4.66	3.91	0.72	6.76
**Chr_10_sg_C_439213_G**	**10 sg C**	**439213**	**A/G**												4.15	3.43	-0.63	8.82
**Chr_11_sg_C_11719181_C**	**11 sg C**	**11719181**	**T/C**				7.4	6.55	1.17	21.02	5.26	4.48	0.86	16.3				
**Chr_2_sg_E_32049720_G**	**2 sg E**	**32049720**	**C/G**	5.73	2.52	21.64	4.07	3.35	0.59	6.27	3.77	3.07	0.51	6.67	11.3	10.35	0.9	19.4
**Chr_2_sg_E_32049728_G**	**2 sg E**	**32049728**	**A/G**	5.83	2.21	21.17												
**Chr_5_sg_E_19112445_C**	**5 sg E**	**19112445**	**T/C**				3.98	3.27	0.77	9.97	4.59	3.84	0.66	10.5				
**Chr_7_sg_E_13418072_C**	**7 sg E**	**13418072**	**T/C**								3.95	3.24	-0.57	7.64	4.71	3.96	-0.65	9.29

^1^The name of the QTNs is based on its position in the genome of C. arabica Et039 genome and on the allele associated with resistance to BHB.

^2^chromosome and subgenome (sg).

^3^QTN positions.

^4^r^2^(%), phenotypic variation of traits explained by each QTN.

**Figure 4:**
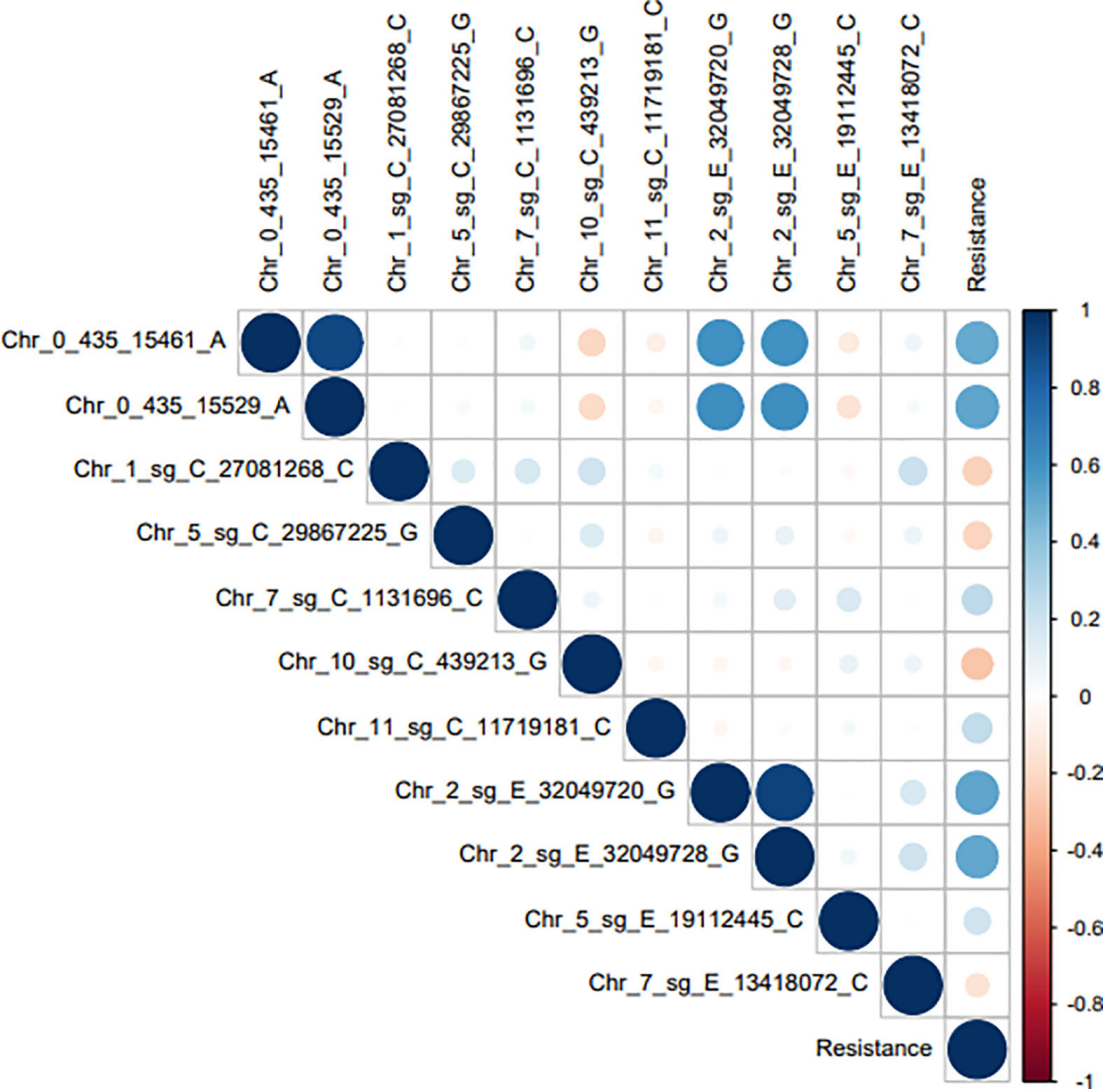
Correlation between the 11 QTNs identified in the GWAS and the resistance to BHB phenotype. The closer to zero, the lower the correlation, positive (blue) or negative (red), which depends on the associated QTN effect.

The QTNs Chr_2_sg_E_32049720_G, Chr_2_sg_E_32049728_G, Chr_0_434_435_15461_A, and Chr_0_434_435_15529_A have the highest association -log10 (p-value) and a high correlation with resistance to BHB (**Table 1**, **Figure 4**). While the other QTNs, identified only through the multi-locus models: Chr_1_sg_C_27081268_C, Chr_5_sg_C_29867225_G, Chr_7_sg_C_1131696_C, Chr_10_sg_C_439213_G, Chr_11_sg_C_11719181_C, Chr_5_sg_E_19112445_C, and Chr_7_sg_E_13418072_C showed a lower correlation to the level of resistance to BHB (**Table 1**, **Figure 4**).

Furthermore, only the IPR 102 cultivar and some Ethiopian accessions like E287 with high resistance to BHB have the QTNs: Chr_2_sg_E_32049720_G, Chr_2_sg_E_32049728_G, Chr_0_434_435_15461_A, and Chr_0_434_435_15529_A (**Supplementary Table 3**).

### Identification of candidate genes

From the *C. arabica* Et039 functional annotation, a total of 31 *C. arabica* genes were identified near to 11 QTNs associated with resistance to BHB (**Supplementary Table 4**). To consider the gene linked to the QTN, distances approximately based on the LD decay result, were used. The LD decay at *r* ^2^ = 0.2 was 66,291 bp (**Supplementary Figure 3**). No genes were identified near to the QTNs Chr_11_sg_C_11719181_C and Chr_5_sg_E_19112445_C. Among the *C. arabica* genes identified near to the QTNs associated with resistance to BHB, we found eight genes with functional annotation related to response to biotic stress described in the literature (**Table 2**).

**Table 2:** Functional annotation of genes near to QTNs associated with resistance to BHB in *C. arabica* identified in this study.

gene^1^	QTN	gene position in relation to the QTN^2^	functional annotation^3^
*g000*	Chr_0_435_15461_A; Chr_0_435_15529_A	(-)12.090; (-)12.158	Serine/threonine-protein kinase D6PKL2
*g009091*	Chr_1_sg_C_27081268_C	(-)30.124	probable carbohydrate esterase At4g34215
*g090102*	Chr_1_sg_C_27081268_C	(+)64.608	Arabinogalactan 1
*g009893*	Chr_5_sg_C_29867225_G	(-)66.800	UBP1-associated protein 2C
*g009954*	Chr_5_sg_C_29867225_G	(-)34.821	S-norcoclaurine synthase-like
*g000156*	Chr_10_sg_C_439213_G	(-)52.180	serine threonine- kinase endoribonuclease IRE1a-like
*g001147*	Chr_10_sg_C_439213_G	(-)49.324	glucan endo-1|3-beta-glucosidase 12-like
*g010741*	Chr_2_sg_E_32049720_G; Chr_2_sg_E_32049728_G	(+)76.156; (+)76.148	Disease resistance protein RPP13 1

^1^The name of the genes was retrieved from the C. arabica Et039 genome functional annotation.

^2^(+) up or (-) downstream position in bp.

^3^The genes functional annotation was retrieved from the C. arabica Et039 genome functional annotation.

Interestingly, near to the QTN with the highest -log10(p-value) of association, Chr_2_sg_E_32049720_G, we found the *g010741* gene with functional annotation for a disease resistance RPP13. This protein is a NBS-LRR, which acts on specific recognition of pathogen and hypersensitivity response (HR). The QTN Chr_0_435_15529_A, also with high -log10(p-value) of association, is linked to a serine threonine- kinase D6PKL2. Another protein of this class, a serine threonine- kinase endoribonuclease IRE1a-like, was identified near to the QTN Chr_10_sg_C_439213_G.

Regarding defense compounds synthesized by plants, we identified *C. arabica* genes with functional annotation for hydrolase (*g009091*), glycosidase (*g001147*), RNA-binding protein linked to callose deposition (*g009946*), and enzyme involved in alkaloid biosynthesis (*g009954*). It is worth mentioning that the QTN Chr_1_sg_C_27081268_C is near to *g090102*, a gene with functional annotation for the Arabinogalactan protein family.

The detailed descriptions of the genes roles in the plants defense mechanism against biotic stresses, as well as their respective literary references, are presented in discussion topic.

### Transcriptional profile of candidate genes by RT-qPCR

**Figure 5:**
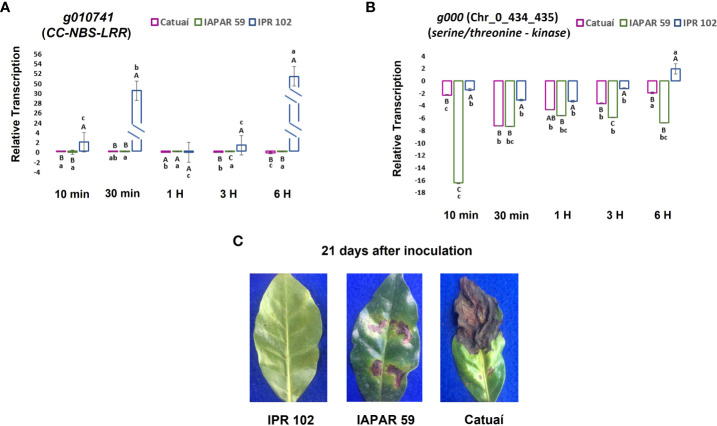
**(A)** Transcription levels of genes related to BHB resistance in leaves of C. *arabica* Catuaí Vermelho IAC 99, IAPAR 59, and IPR 102 inoculated with *P. syringae* pv. *garcae*. **(A)** *Serine/threonine- kinase g000* (Chr_0_434_435) and **(B)** *CC-NBS-LRR g010741* (Chr_2_sg_E). Data represent means ± standard deviation. Upper case letters compare columns of different genotypes at the same tai and lower-case letters compare columns of the same genotype at different tai. Same letters show no difference by the Tukey test (for *P* ≤ 0.05). **(C)** Phenotypic analysis of IPR 102, IAPAR 59, and Catuaí Vermelho IAC 99, 21 days after inoculation.

The genes *g000* (Chr_0_434_435) and *g010741* (Chr_2_sg_E), predicted for encoding serine/threonine-kinase protein and CC-NBS-LRR, respectively, showed no difference in transcription levels in the absence of the pathogen for the Catuaí Vermelho IAC 99, IAPAR 59, and IPR 102 cultivars (**Supplementary Figures 8A, B**). However, after inoculation with the pathogen, different transcription levels were observed (**Figures 5A, B**).

The three cultivars showed repression of *serine/threonine-kinase* transcripts from 10 min (tai). However, the repression in Catuaí Vermelho IAC 99 and IAPAR 59 was higher than observed in IPR 102 during the time course of infection. The cultivar Catuaí Vermelho IAC 99 showed a progressive decrease in repression of *serine/threonine-kinase* transcripts from 30 min to 6h (tai). The cultivar IAPAR 59 also showed a repression decrease at 30 min (tai) but remained stable until 6 h (tai). The *serine/threonine-kinase* transcript repression in IPR 102 remained stable until 6 h (tai) when it showed an upregulation.

Regarding the *CC-NBS-LRR* gene, the highly resistant cultivar IPR 102 showed no change in the level of transcription at 10 min (tai) compared to that observed in the absence of the pathogen, but at 30 min (tai) an upregulation peak approximately 30 times greater than that found in the cultivar in the absence of the pathogen was observed. However, at 1 h and 3 h (tai) the IPR 102 returned to show the same level of transcription that was found in the absence of the pathogen for the *CC-NBS-LRR* gene. The IPR 102 showed another peak of upregulation at 6 h (tai), approximately 50 times higher than the level found in the absence of the pathogen and with statistical difference from the first peak.

The cultivar Catuaí Vermelho IAC 99 showed a pattern of progressive downregulation between 10 min (tai) and 6 h (tai) for *CC-NBS-LRR* transcripts. The cultivar IAPAR 59 also showed a pattern of downregulation of this gene from 10 min (tai), but similar to the serine/threonine-kinase the level of repression remained stable until 6 h (tai).

For the RT-qPCR analyses, the choice of collection times is based on the results of Cheval et al. (2013) and Nobori et al. (2020). According to these authors, the interaction of *Arabidopsis thaliana* vs. *P. syringae* in the early stages, before 6 h (tai), are essential to determine the plant resistance or susceptibility.

All seedlings of Catuaí Vermelho IAC 99, IAPAR 59, and IPR 102 used for qPCR analyses, showed leaves with the score 4 (susceptible), 2 (moderately resistant), and 0 (highly resistant) at 21 (dai), respectively (**Figure 5C**).

### Haplotype analyses

**Figure 6:**
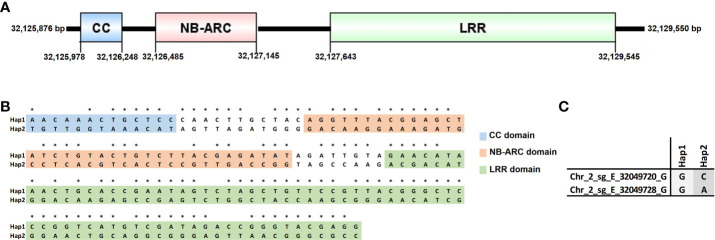
Haplotype analysis. **(A)** The C. *arabica* Et039 *g010741* gene (Chr2_sg_E) and their conserved domains with respective positions in pb. **(B)** The 143 SNPs identified between haplotype 1 (Hap1 – Et039) and haplotype 2 (Hap2 – Caturra and Geisha); and the alleles that compose the haplotypes. The SNPs identified between Hap1 and Hap2, inserted in conserved domains, are highlighted. The position of each SNP identified in the haplotype analysis, in the C. *arabica* Et039 chromosome 2 eugenioides subgenome, is available in **Supplementary Material 2**. The 105 nsSNPs are identified with *. The amino acid change corresponding to each nsSNPs is available in **Supplementary Material 3**. **(C)** The alleles identified in the QTNs Chr_2_sg_E_32049720_G and Chr_2_sg_E_32049728_G in *C*. *arabica* Et039 (Hap1) and in Caturra e Geisha (Hap2).

The *C. arabica* Et039 *g010741* gene showed homology with the genomic region 31,924,108 bp – 31,927,645 bp (Chr2_sg_E) of Caturra, and with the genomic region 1,417,586 bp – 1,421,123 bp (Scaffold_627 | HRSCAF=12830) of Geisha genotype. The homologous genomic region identified in Caturra, corresponds to the locus LOC113732450, and in Geisha to the locus Os08g43010. Both LOC113732450 and Os08g43010 have functional annotation for disease resistance RPP13-like protein 1. From the multiple alignment between *g010741* (Et039), LOC113732450 (Caturra) and Os08g43010 (Geisha), it was possible to identify 143 SNPs in a sequence region of 3,412 bp. Caturra and Geisha showed the same alleles for these 143 SNPs. The position of each SNP identified in the haplotype analysis is available in **Supplementary Material 2**. Thus, the alleles identified in Et039 compose the haplotype 1 (Hap1) and the alleles identified in Caturra and Geisha compose the haplotype 2 (Hap 2) (**Figure 6B**). Among the 143 SNPs identified, 105 correspond to nsSNPs (**Figure 6B**). The amino acid change corresponding to each nsSNPs is available in **Supplementary Material 3**.

From the protein sequence predicted by the gene *g010741*, it was possible to identify the conserved domains N-terminal coiled-coil (ID IPR041118), NB-ARC (ID IPR002182), and LRR (ID IPR032675). The positions, in bp, of the conserved domains identified for this gene in the *C. arabica* Et039 chromosome 2 eugenioides subgenome, was represented in **Figure 6A**. Among the 143 SNPs that compose the haplotypes, 13 are inserted in the CC domain, 37 in the NB-ARC domain and 71 in the LRR domain (**Figure 6B**). Among the 105 nsSNPs, 7 are inserted in the CC domain, 24 in the NB-ARC domain and 60 in the LRR domain (**Figure 6B**).

*C. arabica* Et039, which represents Hap1, showed the alternative allele G both for the QTN Chr_2_sg_E_32049720_G and for the QTN Chr_2_sg_E_32049728_G. Caturra and Gueisha, which represents Hap2, showed the standard allele C for the QTN Chr_2_sg_E_32049720_G and the standard allele A for the QTN Chr_2_sg_E_32049728_G (**Figure 6C**). In the Caturra genome the QTNs Chr_2_sg_E_32049720_G and Chr_2_sg_E_32049728_G were located in Chr2_sg_E at the positions 31,851,359 bp and 31,851,367 bp, respectively. In the Geisha genome the QTNs Chr_2_sg_E_32049720_G and Chr_2_sg_E_32049728_G were located in Scaffold_627|HRSCAF=12830 at the positions 1,493,868 bp and 1,493,859 bp, respectively.

## Discussion

### Gwas

In this study, we identify 11 QTNs associated with resistance to BHB in *C. arabica*. Using a multi-locus method, in relation to the single-locus, we identified a greater number of QTNs associated with response to BHB, with a small effect. The advantages of the multi-locus method over single-locus has already been described in other plants and animal species, such as cotton, maize, and pigs (Li et al., 2018; Su et al., 2018; Xu et al., 2018; Ding et al., 2019). This is possible due to the nature of the multi-locus method, which makes a genome multidimensional scan and a simultaneous estimate of the effect of all SNPs (Cui et al., 2018). The ability to identify a greater number of QTNs and QTNs with small effect associated with the trait of interest increase our understanding of the genetic control of complex traits and the estimation of heritability (Ding et al., 2019; Zhang et al., 2019).

Some multi-locus methods were able to overlap the genomic regions associated with resistance to BHB identified through the single-locus method. The QTN Chr_0_435_15529_A was detected using the single-locus MLM and through the multi-locus mrMLM. A complementarity between the results of the single-locus and multi-locus methods was also found for the QTN Chr_2_sg_E_32049720_G, which was identified through the single-locus MLM and the multi-locus mrMLM, FASTmrMLM, and, ISIS EM-BLASSO. Other authors have observed the same ability of multi-locus methods to overlap part of the results obtained by single-locus methods (Misra et al., 2017; Li et al., 2018).

It is worth mentioning that the multi-locus method considers the LD and decreases the number of associated QTNs in the same haplotype (Ding et al., 2019). Probably because of this, the QTN Chr_2_sg_E_32049728_G identified by MLM was not identified in the mrMLM, FASTmrMLM, and ISIS EM-BLASSO, since it is very close to the QTN Chr_2_sg_E_32049720_G. The same was observed for the QTN Chr_0_435_15461_A, in relation to the QTN Chr_0_435_15529_A, in the result of the mrMLM.

Some QTNs were identified through different multi-locus methods, such as the QTN Chr_2_sg_E_32049720_G which was identified by the mrMLM, FASTmrMLM, and ISIS EM-BLASSO, the QTNs Chr_11_sg_C_11719181_C and Chr_5_sg_E_19112445_C identified by the mrMLM, and FASTmrMLM, the QTN Chr_7_sg_C_1131696_C identified by the mrMLM, and ISIS EM-BLASSO, and the QTN Chr_7_sg_E_13418072_C identified by the FASTmrMLM, and ISIS EM-BLASSO, thus, demonstrating the robustness of the association methods as well the reliability of the QTNs.

### Identification of candidate genes related to plant defense to BHB

Plant diseases threaten plant production and, consequently, world food security. Identification and understanding of the mechanisms and genetic bases of resistance is essential to achieve greater and more sustainable production. Genes involved in resistance, once identified and validated, can be used to improve the crop based on molecular assisted breeding or using genome editing tools.

Plant resistance is based on metabolites and proteins that directly suppress pathogen development, leading to reduced susceptibility. These metabolites and proteins can be constitutively present or synthesized in plants following pathogen perception (Kushalappa et al., 2016). After the plant proteins recognize the pathogen a hierarchy of downstream genes such as phytohormones (salicylic acid, jasmonic acid, and ethylene), mitogen-activated protein kinases (MAPKs), and transcription factors (TFs), which regulate downstream genes that produce defense metabolites (phytoanticipins, phytoalexins, and complex conjugates that are deposited to reinforce cell walls), and proteins (pathogenesis-related proteins, detoxify toxins proteins, and enforcing cell wall proteins) (Kushalappa and Gunnaiah, 2013).

Some *C. arabica* genes, near to the QTNs associated with resistance to BHB, identified in this work, have functional annotation for proteins that play roles in the plants defense mechanism against pathogens.

The gene *g010741*(Chr_2_sg_E) is ortholog to the RPP13, a downy mildew resistance protein (NBS-LRR) originally identified in the Niederzenz (Nd-1) ecotype of *Arabidopsis* (Bittner-Eddy et al., 2000). Furthermore, Lewis et al. (2013) identified that RPP13L4/ZAR1 is dependent on a nonfunctional kinase, which functions as a “decoy”, for pathogen recognition.

Other genes that act as defense signal receptor and transmitter proteins were also identified. The gene *g000* (Chr0) has a functional annotation for serine/threonine-protein kinase D6PKL2. In Zhang et al. (2021) this protein was described as specifically expressed after *Fusarium oxysporum* infection in *Vernicia montana* resistant trees. Furthermore, transgenic analysis in *Arabidopsis* and tomato revealed that D6PKL2 significantly enhanced resistance in both species, whereas the *d6pkl2* mutant displayed reduced resistance against *F. oxysporum* (Zhang et al., 2021). The gene *g000156* (Chr_10_sg_C) has orthology with an Arabidopsis gene that encondes a serine/threonine-kinase endoribonuclease IRE1a-like protein. In *Arabidopsis*, this gene was transcriptionally induced during bacterial pathogen *P. syringae* pv. *maculicola* infection and plays a predominant role in the secretion of pathogenesis-related proteins during salicylic acid treatment. *Arabidopsis* mutant plants without this gene showed enhanced susceptibility to a bacterial pathogen and were deficient in establishing systemic acquired resistance (SAR) (Moreno et al., 2012).

A protein with hydrolytic function, the probable carbohydrate esterase At4g34215, was identified in the *g009091* (Chr_1_sg_C) gene functional annotation. Balmant et al. (2015) reported that a carbohydrate esterase exhibited a significant fold change in redox modification and a significant fold change in transcriptional level, during the interaction between tomato resistant genotype (PtoR) and *P*. *syringae* pv *tomato*. The gene *g001147* (Chr10_C) also showed functional annotation for a protein with hydrolytic function, a glucan endo-1|3-beta-glucosidase. This enzyme are grouped in the PR-2 family of pathogenesis-related proteins and catalyze β-1,3-glucans cell wall structural component of fungal and bacteria (Bachman and McClay, 1996; Balasubramanian et al., 2012).

Regarding secondary metabolites of plant defense, the functional annotation of the *g009954* (Chr_5_sg_C) gene corresponds to an S-norcoclaurine synthase-like. S-norcoclaurine is the ultimate precursor to benzylisoquinoline, an alkaloids that plays a role in the plants defense against herbivores and pathogens (Liscombe et al., 2005; Yogendra et al., 2017).

The *g009893* (Chr_5_sg_C) has functional annotation for a UBP1-associated protein 2C. This protein is a RNA-binding and provides hints to another level of regulation posttranscriptionally. In Bove et al. (2008) is reported that transcripts levels of UBA2 increased following mechanical wounding. In another work, using transient expression, Kim et al. (2008) identified that *Arabidopsis* UBA2 proteins in *Nicotiana benthamiana* leaves induces a programmed-cell-death/senescing phenotype, while constitutive expression causes an early lethality phenotype in *Arabidopsis*.

The gene *g090102* (Chr1_C) has functional annotation for Arabinogalatans proteins family. Arabinogalactans proteins (AGPs) are structural polysaccharides associated with proteins of the plant cell wall. Plants can secrete and accumulate AGPs at infection sites, creating cross-links with pectin. Pathogens can secret hydrolytic enzymes that damage AGPs. In this way, the AGPs degradation, by the pathogen’s hydrolytic enzymes action, can elicit plant defense response through damage-associated molecular patterns (DAMPs) (Villa-Rivera et al., 2021).

### Identification of genomic regions probably involved in qualitative resistance

Major genes that recognize the pathogen and trigger PTI and ETI, resulting in HR, are essential to promote qualitative resistance (Jones and Dangl, 2006; Yuan et al., 2021). In the absence of these genes, other genes with minor effects can promote partial resistance to the pathogen and a decrease in symptom severity (St. Clair, 2010). This partial resistance to the pathogen corresponds to quantitative resistance and produces a continuous distribution of phenotypes between the susceptible and the resistant plant (Corwin and Kliebenstein, 2017).

The elucidation of which genes act on qualitative, quantitative, or both types of resistance is important, since the combination of qualitative and quantitative resistance, in the same genotype, is considered a promising strategy in breeding programs (Pilet-Nayel et al., 2017). This is because quantitative resistance can exercise different selection pressures on the pathogen (Quenouille et al., 2014). In addition, it can reduce the effective population size of the pathogen, which increases the genetic drift and delays the emergence of new virulent forms (Brun et al., 2010). Thus, the effectiveness of resistance conferred by major genes is preserved.

The QTNs Chr_2_sg_E_32049720_G, Chr_2_sg_E_32049728_G, Chr_0_434_435_15461_A, and Chr_0_434_435_15529_A can be associated with qualitative resistance to BHB. Four of our results reinforces this hypothesis. Firstly, the QTNs showed a strong correlation with the resistance phenotype (**Figure 4**). Secondly, only *C. arabica* plants with these 4 QTNs showed qualitative resistance to BHB (**Supplementary Table 3**). Thirdly, the genes close to these QTNs are predicted to encode a serine/threonine-kinase protein and a CC-NBS-LRR protein, which act *via* an indirect interaction with the pathogen effector (**Table 2**). Finally, fourth, those genes showed a very similar transcriptional profile for cultivars Catuaí Vermelho IAC 99 (susceptible) and IAPAR 59 (quantitative resistance), while the cultivar IPR 102 (qualitative resistance) showed a different profile (**Figures 5A, B**), with these genes being upregulated in relation to the susceptible cultivars.

In Arabidopsis and tomato interaction with *P. syringae* the effector AvrPto binds to pattern recognition receptors (PRRs), including receptor-like protein kinases (RLKs) protein, and blocks pattern triggered immunity (PTI) activation. This block enhances host susceptibility. In resistant plants, a serine/threonine-kinase can mimic the kinase domain of the RLKs and bind to AvrPto. An NB-LRR protein recognizes the bind between the effector and the serine/threonine-kinase and then activates the effector triggered immunity (ETI). Thus, PTI and ETI are activated, resulting in a hypersensitivity response (HR) (**Supplementary Figure 9**) (Jones and Dangl, 2006; Zipfel and Rathjen, 2008; Zong et al., 2008).

Thus, the genes *g000* (Chr_0_434_435) and *g010741* (Chr_2_sg_E) could probably play a similar role in resistance to BHB in *C. arabica*. These results are an indication that the transcription levels for genes *g000* (Chr_0_434_435) *serine/threonine-kinase* and *g010741*(Chr_2_sg_E) *CC-NBS-LRR*, found in IPR 102, may be necessary to activate qualitative resistance to BHB.

The haplotype analysis performed in this work identified a high number of polymorphisms in the *CC-NBS-LRR* gene, among *C. arabica* genotypes. Interestingly, Et039 (Hap1), showed the alternative alleles associated with resistance to BHB for the QTNs Chr_2_sg_E_32049720_G and Chr_2_sg_E_32049728_G. Meanwhile, Caturra and Geisha (Hap2) showed the standard alleles for the same QTNs (**Figure 6C**). The nsSNP identified between Hap1 and Hap2, mainly those inserted in conserved domains (**Figure 6B**), may be related to the defense response functionality of the gene. However, the haplotypes must be validated in a greater number of genotypes with phenotyping for resistance to BHB in order to reinforce the role of this genes in the resistance to BHB.

### Relationship between the loci involved in resistance to BHB and the *C. arabica* domestication process

The population structure results showed by hierarchical analysis, PCA, and Bayesian approach at K = 3 were consistent for clustering the *C. arabica* Ethiopian accessions and the cultivars according to their domestication level characteristics. The admixed accessions identification was possible with the Bayesian approach. Furthermore, the Bayesian approach at K = 3 was the most suitable in demonstrating the genetic proximity between the accessions collected at the East of the Great Rift Valley and the *C. arabica* cultivars. This result corroborate with other studies that identified the movement of *C. arabica* from West to East of the Great Rift Valley, in direction to Yemen, from where *C. arabica* was disperse worldwide (Montagnon and Bouharmont, 1996; Silvestrini et al., 2007; Scalabrin et al., 2020).

**Figure 7:**
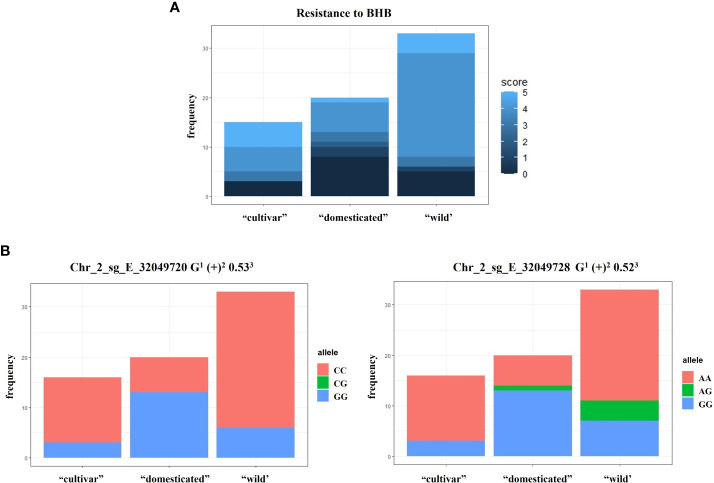
Population structure and relation with resistance to BHB. **(A)** The distribution frequency of accessions resistance to BHB into the “cultivars” (n = 16), “domesticated” (n = 20), and “wild” (n = 33) clusters identified in population structure Bayesian approach at K = 3. C*. arabica* Ethiopian accessions classified as admixed were disregarded in this analysis. The resistance to BHB is based in a scale with a score from 0 to 5, where 0 corresponds to highly resistant plants, 1 to resistant, 2 to moderately resistant, 3 to moderately susceptible, 4 to susceptible, and 5 to highly susceptible. **(B)** The distribution frequency of the QTNs associated with resistance to BHB into the “cultivars”, “domesticated”, and “wild” clusters. QTN associated with resistance to BHB with its respective location in the C. *arabica* Et039 genome^1^; QTN effect^2^; *r^2^* (%) of the QTN in relation to the resistance phenotype^3^.

From the frequency analysis of resistance to BHB into the clusters formed in population structure Bayesian approach at K = 3, it was possible to identify a higher frequency of resistance to BHB in the “domesticated” cluster (**Figure 7A**). However, in the *C. arabica* cultivars, there was a decrease in the frequency of accessions resistant to BHB. The QTNs Chr_2_sg_E_32049720_G and Chr_2_sg_E_32049728_G, with the highest -log10 (p-values) of association identified by both the single-locus and multi-locus methods, showed a higher frequency in the “domesticated” cluster (**Figure 7B**). The distribution frequency of the others QTNs associated with resistance to BHB identified in this work are shown in **Supplementary Figure 7**.

The highest frequency, in the “domesticated” cluster, of *C. arabica* accessions resistant to BHB and of the QTNs Chr_2_sg_E_32049720_G and Chr_2_sg_E_32049728_G, suggest that resistance to BHB is a trait inserted in loci that may have had an influence in plants selection during the *C. arabica* domestication history (**Figures 7A, B**). The influence of genomic regions linked to resistance to disease in the domestication process has already been described for other species, such as peanut (*Arachis hypogaea*), tomato (*Solanum pimpinellifolium* L. and *S. lycopersicum* L.), and soybean (*Glycine max* L.) (Zhao et al., 2015; Zhang et al., 2017; Razifard et al., 2020). In addition, genomic regions of resistance to disease showed the highest selection rate, during the divergence between *Coffea arabica*, *C. canephora*, and *C. excelsa* (Huang et al., 2020).

However, the lower frequency in the “cultivar” cluster of both *C. arabica* resistant to BHB and QTNs with the highest -log10 (p-values) identified in this GWAS agrees with the low genetic variability and low resistance to pathogens known in *C. arabica* cultivars (van der Vossen, 1985; Anthony et al., 2002). The lack of resistance to diseases in cultivars, a disadvantage in relation to the diversity found in wild genotypes, is extensively reported in the literature in many different species (Lam et al., 2011; Stalker et al., 2013; Cao et al., 2014). According to these studies, characteristics such as greater palatability, greater production, and fruit size, had greater relevance in the cultivar development over resistance to diseases. In the case of *C. arabica*, most cultivars are derived from only two varieties: Typica and Bourbon (Krug et al., 1939). This fact makes the genetic diversity among commercial *C. arabica* cultivars quite limited (Anthony et al., 2002; Steiger et al., 2002; Vidal et al., 2010). In addition, breeding programs that originated these cultivars prioritized criteria such as high productivity, beverage quality, and drought tolerance (van der Vossen, 1985). As a result, a large genetic bottleneck was formed. It is important to observe that, in this work, the haplotype analysis of a *CC-NBS-LRR* gene showed a greater genetic diversity from an Ethiopian accession (*C. arabica* Et039) than in commercial genotypes (Caturra and Geisha).

These results reinforce the importance of conservation of *C. arabica* wild types, both in their native habitats and in germplasm banks. Since their genetic diversity can be used to identify genes involved in traits of interest, such as resistance to diseases, and to elucidate the *C. arabica* domestication process.

## Conclusions

This study provides 11 QTNs associated with resistance to BHB that have potential for application in Marker Assisted Selection and the characterization of a serine/threonine-kinase and a CC-NBS-LRR, probably, involved in qualitative resistance to BHB, could help to direct the breeding and could be a gene editing target.

Our results demonstrate that the combination of different GWAS methods can increase the efficiency and accuracy of obtaining a panel of QTNs associated with features of interest.

Furthermore, this work provides an insight into the relationship between the loci involved in resistance to BHB and the *C. arabica* domestication history. This reinforces the importance of maintenance of the Ethiopian coffee collections as a genetic diversity source for breeding programs on the pathway to achieve sustainable agriculture.

This is the first report for the interaction between *C. arabica* and *P. syringae* at the genetics and molecular level. In this way, the results presented are a starting point for future works that may bring greater evidence and validation of the resistance genes identified, such as gene expression analysis and validation in gene knock out mutants or gene overexpressing mutants.

## Data availability statement

The GBS data presented in the study are deposited in the National Center for Biotechnology Information repository, accession number PRJNA882422.

## Author contributions

CA, LP wrote the manuscript. CA, GCS, LFPP designed the experiment. MSF, RVF, LMN, BSS performed genotyping data. CA, GHS, LMRR, SALD participated in preparation of the materials and phenotype analysis. DSD, LFPP, MLVR provide funds for research. All the authors read, contributed and approved the final version of the manuscript. All authors contributed to the article and approved the submitted version.

## Funding

This work was supported by grants from the Consórcio Pesquisa Café (Grant 10.18.20.027.00), INCT Café and FAPEMIG. CA, RVF, LMN, BSS and MSF received a PhD fellowship from CAPES (Finance Code 001). LP and DD received a Research Fellowship from CNPq (311452/2020-5).

## Acknowledgments

We gratefully acknowledge the Arabica Coffee Genome Consortium (ACGC) for providing access to *C. arabica* Et039 genome data. We also acknowledge the Consórcio Pesquisa Café, INCT Café, Fundação de Amparo à Pesquisa do Estado de Minas Gerais (FAPEMIG), the Conselho Nacional de Desenvolvimento Científico e Tecnológico (CNPq), and the Coordenação de Aperfeiçoamento de Pessoal de Nível Superior (CAPES) for financial support and fellowships.

## Conflict of interest

GCS was employed by company Tropical Melhoramento & Genética (TMG).

The remaining authors declare that the research was conducted in the absence of any commercial or financial relationships that could be construed as a potential conflict of interest.

## Publisher’s note

All claims expressed in this article are solely those of the authors and do not necessarily represent those of their affiliated organizations, or those of the publisher, the editors and the reviewers. Any product that may be evaluated in this article, or claim that may be made by its manufacturer, is not guaranteed or endorsed by the publisher.

## Supplementary material

Supplementary Figure 1 Histogram of the disease distribution, values of response to Bacterial Halo Blight obtained in field evaluation (Mohan et al., 1978; Ito et al., 2008). The X-axis represents the classes of distribution for the 120 *C. arabica* wild accessions (blue), 11 *C. arabica* cultivars (red) and BA-10 genotype evaluated. The Y-axis shows the count of *C. arabica* genotypes in each category.

Supplementary Figure 2 Distribution of 11,290 SNPs, identified in 120*C. arabica* wild accessions, 11 *C. arabica* cultivars and BA-10 genotype, in the *C. arabica* genome. Each chromosome has its size information in base pairs. The bars correspond to the number of SNPs on each chromosome.

Supplementary Figure 3 Linkage disequilibrium (LD) decay for 16,146 SNPs obtained after quality filters and the removal of SNPs from Chr zero. Each point represents a physical distance (bp) between SNPs pairs and their squared correlation coefficient (r2). The red line represents the line for non-linear regression against physical distance. The green line represents the LD decay value for r2 = 0.2.

Supplementary Figure 4 Dissimilarity matrix representing the genetic distance between the 120 *C. arabica* wild accessions, 11 *C. arabica* cultivars and BA-10 genotype. The color intensity is proportional to the value of genetic distance between the accessions. The matrix was constructed based on the 11,290 SNPs identified in this population. The color intensity is proportional to the value of genetic distance between the accessions.

Supplementary Figure 5 Structure analysis of 120 *C. arabica* wild accessions, 11 *C. arabica* cultivars and BA-10 genotype. Evolution of ΔK values (y-axis) according to the number of genetic groups (x-axis).

Supplementary Figure 6 Classification of 132 accessions of *C. arabica*, using 11,290 SNPs identified in this population, into K = 2, K = 3, K = 4, and K = 5 using STRUCTURE 2.3.4. The distribution of the accessions to different populations is indicated by the color code. Numbers on the y-axis show the subgroup membership, and the x-axis shows the different accessions.

Supplementary Figure 7 Population structure and relation with resistance to BHB. The distribution frequency of the QTNs associated with resistance to BHB into the “cultivars” (n = 16), “domesticated” (n = 20), and “wild” (n = 33) clusters identified in population structure Bayesian approach at K = 3. *C. arabica* Ethiopian accessions classified as admixed were disregarded in this analysis. QTN associated with resistance to BHB with its respective location in the *C. arabica* Et039 genome1; QTN effect2; r2 (%) of the QTN in relation to the resistance phenotype3.

Supplementary Figure 8 Transcribed levels in the absence of the pathogen. **(A)** The g000 (Chr_0_434_435) and **(B)** g010741 (Chr_2_sg_E). Data represent the means ±  standard deviation. Upper case letters compare columns of different genotypes. Same letters show no difference by Tukey test (for P ≤ 0.05).

Supplementary Figure 9 Model for PTI-inhibition and ETI-induction by *Pseudomonas syringae* effector. In susceptible plants, the effectors bind to PRRs. This blocks PTI activation and enhances host susceptibility to the bacterium. In resistant plants, serine/threonine – kinase is evolved to mimic the kinase domain of PRRs and binds to the effectors to trigger ETI, resulting in a resistance outcome. Blocking **(A)** and triggering **(B)** a hypersensitive response by *Pseudomonas syringae* effector.
