# Supplementary material for: Genome-wide association study for resistance to Pseudomonas syringae pv. garcae in Coffea arabica
Source: Front Plant Sci. 2022 Oct 18;13:989847. doi: 10.3389/fpls.2022.989847 (PMC9624508; doi:10.3389/fpls.2022.989847)
Supplement: Supplementary Figure 1 — Histogram of the disease distribution, values of response to Bacterial Halo Blight obtained in field evaluation (Mohan et al., 1978; Ito et al., 2008). The X-axis represents the classes of distribution for the 120 C. arabica wild accessions (blue), 11 C. arabica cultivars (red) and BA-10 genotype evaluated. The Y-axis shows the count of C. arabica genotypes in each category. [file DataSheet_1.zip › Supplementary Material 1.DOCX]

**Supplementary Material 1.** The multiple alignment between The *C. arabica* Et039 *g010741* gene (Chr2_sg_E, position 32,109,397 bp – 32,111,166 bp), the homologous genomic region (Chr2_sg_E, position 31,924,108 bp – 31,927,645 bp) of Caturra, and the homologous genomic region (Scaffold_627 | HRSCAF=128301, position 417,586 bp – 1,421,123 bp) of Geisha. The genomic sequence of 3,412 bp used to identified SNPs is highlighted in blue.

Et039 GGCTCTCTCTTTCTCTATCTTTCTCTGGTTTCCTTGTGCAAGCTTCTTTTGCTTTTACCT

Caturra ------------------------------------------------------------

Geisha ------------------------------------------------------------

Et039 TGGCCTGGAAAAATGGCTCTTGCATTAGTCGGAGGCTCTTTTCTCTCTGCTTTCCTTCAA

Caturra ------------ATGGCTCTTGCATCAGTCGGAGGCTCTCTTCTCTCTGCTTTCCTTCAA

Geisha ------------------------------------------------------------

Et039 GTGCTGTTTGATAGGATGGCGTCCCCAGAGTTCCTGAATTTGTTTCGTAAGCGAAAAGCT

Caturra GTGCTGTTTGATAGGATGGCGTCCCCAGAGTTCCTGAATTTGTTTCGTAAGCGAAAAGCT

Geisha ------------------------------------------------------------

Et039 GATGATGAACTCCTCAAGAAGCTCAAGA**A**CAACTT**A**CTGGCAGTTGGAGCAGTGCTTGAT

Caturra GATGATGAGCTCCTCAAGAAGCTCAAGA**T**CAACTT**G**CTGGCAGTTGGAGCAGTGCTTGAT

Geisha ------------------AAGCTCAAGA**T**CAACTT**G**CTGGCAGTTGGAGCAGTGCTTGAT

********** ****** ************************

Et039 GA**C**GC**A**GA**A**AACAAGGAAATAAGCA**A**CCAAGC**C**GTCAAGGAATGGCTTG**TG**GAG**C**TTCAT

Caturra GA**T**GC**T**GA**G**AACAAGGAAATAAGCA**G**CCAAGC**T**GTCAAGGAATGGCTTG**AA**GAG**A**TTCAT

Geisha GA**T**GC**T**GA**G**AACAAGGAAATAAGCA**G**CCAAGC**T**GTCAAGGAATGGCTTG**AA**GAG**A**TTCAT

** ** ** **************** ****** **************** *** *****

Et039 GAAATTGTTTACCAGGCAGATGATTTGCTCGATGAGATCAACACTGAAG**T**TCTGCGAGTT

Caturra GAAATTGTTTACCAGGCAGATGATTTGCTCGATGAGATCAACACTGAAG**C**TCTGCGAGTT

Geisha GAAATTGTTTACCAGGCAGATGATTTGCTCGATGAGATCAACACTGAAG**C**TCTGCGAGTT

************************************************* **********

Et039 **C**AGGTAGAATC**C**GAGTAC**C**AAAGCTCAACCA**A**C**A**TCCTGG**C**AAGTGCTTCCACTTAT**T**TT

Caturra **A**AGGTAGAATC**T**GAGTAC**A**AAAGCTCAACCA**G**C**T**TCCTGG**T**AAGTGCTTCCACTTAT**A**TT

Geisha **A**AGGTAGAATC**T**GAGTAC**A**AAAGCTCAACCA**G**C**T**TCCTGG**T**AAGTGCTTCCACTTAT**A**TT

********** ****** ************ * ****** **************** **

Et039 TCATCTTTCAGTAATCAGTTCTTTAAAAGGATTATGCCTGA**T**ATAGAAAAAGTTGTGATT

Caturra TCATCTTTCAGTAATCAGTTCTTTAAAAGGATTATGCCTGA**G**ATAGAAAAAGTTGTGATT

Geisha TCATCTTTCAGTAATCAGTTCTTTAAAAGGATTATGCCTGA**G**ATAGAAAAAGTTGTGATT

***************************************** ******************

Et039 AGCCT**G**GAGGGATTTATACAGCAAATCAATCCCTTAGGTTTGCAAGTTGTTGAACCGAAA

Caturra AGCCT**A**GAGGGATTTATACAGCAAATCAATCCCTTAGGTTTGCAAGTTGTTGAACCGAAA

Geisha AGCCT**A**GAGGGATTTATACAGCAAATCAATCCCTTAGGTTTGCAAGTTGTTGAACCGAAA

***** ******************************************************

Et039 ATACGATCATATCGACTGCCTTCGACTTCTTTGGTCGATGAGGA**CT**CTGT**T**TATGGTAGA

Caturra ATACGATCATATCGACTGCCTTCGACTTCTTTGGTCGATGAGGA**TG**CTGT**G**TATGGTAGA

Geisha ATACGATCATATCGACTGCCTTCGACTTCTTTGGTCGATGAGGA**TG**CTGT**G**TATGGTAGA

******************************************** **** *********

Et039 GAT**A**TTGA**CA**AGGA**G**AA**G**ATA**T**TCCAA**T**TGTTGTTGTC**T**GAGGAT**A**A**C**A**G**GGGAGAC**G**AT

Caturra GAT**G**TTGA**GG**AGGA**A**AA**C**ATA**A**TCCAA**A**TGTTGTTGTC**G**GAGGAT**G**A**A**A**A**GGGAGAC**A**AT

Geisha GAT**G**TTGA**GG**AGGA**A**AA**C**ATA**A**TCCAA**A**TGTTGTTGTC**G**GAGGAT**G**A**A**A**A**GGGAGAC**A**AT

*** **** **** ** *** ***** ********** ****** * * ******* **

Et039 **A**TC**G**CTGTAGTT**C**CAATAGT**T**GG**A**CAAGGTGGGATTGGTAAGACTACCTTGG**TC**CAATTG

Caturra **G**TC**A**CTGTAGTT**T**CAATAGT**G**GG**C**CAAGGTGGGATTGGTAAGACTACCTTGG**CT**CAATTG

Geisha **G**TC**A**CTGTAGTT**T**CAATAGT**G**GG**C**CAAGGTGGGATTGGTAAGACTACCTTGG**CT**CAATTG

** ******** ******* ** **************************** ******

Et039 GTTTACAATGATAAGAGGGTGAAGAAT**T**ATTTCCCTACCAAGGCATGGGTTTGTGTATCA

Caturra GTTTACAATGATAAGAGGGTGAAGAAT**C**ATTTCCCTACCAAGGCATGGGTTTGTGTATCA

Geisha GTTTACAATGATAAGAGGGTGAAGAAT**C**ATTTCCCTACCAAGGCATGGGTTTGTGTATCA

*************************** ********************************

Et039 GAAGAGTAT**G**ATGC**T**ACCAGGATAAC**A**AAGGAACTCCTTAGGGAA**C**T**T**G**G**CAT**T**TCATT**C**

Caturra GAAGAGTAT**A**ATGC**C**ACCAGGATAAC**G**AAGGAACTCCTTAGGGAA**T**T**C**G**A**CAT**C**TCATT**T**

Geisha GAAGAGTAT**A**ATGC**C**ACCAGGATAAC**G**AAGGAACTCCTTAGGGAA**T**T**C**G**A**CAT**C**TCATT**T**

********* **** *********** ****************** * * *** *****

Et039 TC**T**GA**T**TCC**A**GTGAGAGTTTGAATTCCCT**C**CAGGTTAAGCTACAACAAGGCCTAACCGAT

Caturra TC**C**GA**C**TCC**G**GTGAGAGTTTGAATTCCCT**T**CAGGTTAAGCTACAACAAGGCCTAACCGAT

Geisha TC**C**GA**C**TCC**G**GTGAGAGTTTGAATTCCCT**T**CAGGTTAAGCTACAACAAGGCCTAACCGAT

** ** *** ******************* ******************************

Et039 AAAAAGTTTCTTCTTGTACTGGATGATGTTTGGAATGATGACTACGATGACTGG**G**ATAAA

Caturra AAAAAGTTTCTTCTTGTACTGGATGATGTTTGGAATGATGACTACGATGACTGG**T**ATAAA

Geisha AAAAAGTTTCTTCTTGTACTGGATGATGTTTGGAATGATGACTACGATGACTGG**T**ATAAA

****************************************************** *****

Et039 TTGAAGATGCTGGTCAAAGGTGGTTCTGAGGGAAGTAAGATAATTGTGACAACAAGAGAT

Caturra TTGAAGATGCTGGTCAAAGGTGGTTCTGAGGGAAGTAAGATAATTGTGACAACAAGAGAT

Geisha TTGAAGATGCTGGTCAAAGGTGGTTCTGAGGGAAGTAAGATAATTGTGACAACAAGAGAT

************************************************************

Et039 GAGAGAATTGCACTTATGATG**A**GTC**G**TAAAATGTC**A**ATTCAT**T**ATTTGG**A**TTTGTTATCT

Caturra GAGAGAATTGCACTTATGATG**G**GTC**A**TAAAATGTC**C**ATTCAT**C**ATTTGG**G**TTTGTTATCT

Geisha GAGAGAATTGCACTTATGATG**G**GTC**A**TAAAATGTC**C**ATTCAT**C**ATTTGG**G**TTTGTTATCT

********************* *** ********* ****** ****** **********

Et039 GAGGAGGATTCTTGGGTCTTATTTGAGAAACATGCATTTGGA**T**GCAAAGACAATGAAATA

Caturra GAGGAGGATTCTTGGGTCTTATTTGAGAAACATGCATTTGGA**G**GCAAAGACAATGAAATA

Geisha GAGGAGGATTCTTGGGTCTTATTTGAGAAACATGCATTTGGA**G**GCAAAGACAATGAAATA

****************************************** *****************

Et039 CGGCCAGAACTTGAAGTGATAGGAAAAAAAATTGTGAACAAGTGTGAAGGGTTGCCTTTG

Caturra CGGCCAGAACTTGAAGTGATAGGAAAAAAAATTGTGAACAAGTGTGAAGGGTTGCCTTTG

Geisha CGGCCAGAACTTGAAGTGATAGGAAAAAAAATTGTGAACAAGTGTGAAGGGTTGCCTTTG

************************************************************

Et039 GCTGTGAAAACAATTGCAGGGCTTTTGCGTTCAAGAAGCACGGTTGAGGAGTGGGAAGAG

Caturra GCTGTGAAAACAATTGCAGGGCTTTTGCGTTCAAGAAGCACGGTTGAGGAGTGGGAAGAG

Geisha GCTGTGAAAACAATTGCAGGGCTTTTGCGTTCAAGAAGCACGGTTGAGGAGTGGGAAGAG

************************************************************

Et039 ATTTTAAGAAATGATTTATGGAACCAGAC**A**AGAAATCCGAATGGCATCTTGCCAGCGTTG

Caturra ATTTTAAGAAATGATTTATGGAACCAGAC**T**AGAAATCCGAATGGCATCTTGCCAGCGTTG

Geisha ATTTTAAGAAATGATTTATGGAACCAGAC**T**AGAAATCCGAATGGCATCTTGCCAGCGTTG

***************************** ******************************

Et039 AGATTAAGTTACATGCATCTTCCTTCCCATCTAAAAAG**G**TGCTTTGCTT**A**CTGTGCTGTG

Caturra AGATTAAGTTACATGCATCTTCCTTCCCATCTAAAAAG**A**TGCTTTGCTT**G**CTGTGCTGTG

Geisha AGATTAAGTTACATGCATCTTCCTTCCCATCTAAAAAG**A**TGCTTTGCTT**G**CTGTGCTGTG

************************************** ********** **********

Et039 TTTCATAAAGATTTCTGGTTTTCTAAACAGGAAATAATTCAGTTATGGCATGCTAATGGA

Caturra TTTCATAAAGATTTCTGGTTTTCTAAACAGGAAATAATTCAGTTATGGCATGCTAATGGA

Geisha TTTCATAAAGATTTCTGGTTTTCTAAACAGGAAATAATTCAGTTATGGCATGCTAATGGA

************************************************************

Et039 CTTTTGGAGCACCCAAGAAATAATGAAAGTATTGAAGACATAGGTGGAGTGTACTTGCGG

Caturra CTTTTGGAGCACCCAAGAAATAATGAAAGTATTGAAGACATAGGTGGAGTGTACTTGCGG

Geisha CTTTTGGAGCACCCAAGAAATAATGAAAGTATTGAAGACATAGGTGGAGTGTACTTGCGG

************************************************************

Et039 GAATTGAGATTGAGGTCATTGTTGTGGCAGTCAA**TTG**ACAATACATTCTCTATGCACGAT

Caturra GAATTGAGATTGAGGTCATTGTTGTGGCAGTCAA**CCA**ACAATACATTCTCTATGCACGAT

Geisha GAATTGAGATTGAGGTCATTGTTGTGGCAGTCAA**CCA**ACAATACATTCTCTATGCACGAT

********************************** ***********************

Et039 CTTATCAATGATTTGGCTAGATTTGTTTCTGGAAAATATTGTCTCAGGTTGGAAGATCAT

Caturra CTTATCAATGATTTGGCTAGATTTGTTTCTGGAAAATATTGTCTCAGGTTGGAAGATCAT

Geisha CTTATCAATGATTTGGCTAGATTTGTTTCTGGAAAATATTGTCTCAGGTTGGAAGATCAT

************************************************************

Et039 TACCCGGGCTATGGTACAACAGCTAGTGTACGTAACTTCACATATTATCCTAGCA**T**GTAT

Caturra TACCCGGGCTATGGTACAACAGCTAGTGTACGTAACTTCACATATTATCCTAGCA**A**GTAT

Geisha TACCCGGGCTATGGTACAACAGCTAGTGTACGTAACTTCACATATTATCCTAGCA**A**GTAT

******************************************************* ****

Et039 GACAC**A**TTTGATAAGCTTAAACTCTTGAGGGAGGCCAAGAGTTTAAGAACATTCTATCCA

Caturra GACAC**G**TTTGATAAGCTTAAACTCTTGAGGGAGGCCAAGAGTTTAAGAACATTCTATCCA

Geisha GACAC**G**TTTGATAAGCTTAAACTCTTGAGGGAGGCCAAGAGTTTAAGAACATTCTATCCA

***** ******************************************************

Et039 GTTTGTAGGAGTAACTTTGCTTTC**G**G**A**G**A**TGAAATAATAAGCAACAAATTTTTACATGAT

Caturra GTTTGTAGGAGTAACTTTGCTTTC**A**G**C**G**G**TGAAATAATAAGCAACAAATTTTTACATGAT

Geisha GTTTGTAGGAGTAACTTTGCTTTC**A**G**C**G**G**TGAAATAATAAGCAACAAATTTTTACATGAT

************************ * * *******************************

Et039 GTGTTACCCAGATTCAAGTCCTTAAGGGTTCTATCATTGTACAATCGAAGTATACTTAAG

Caturra GTGTTACCCAGATTCAAGTCCTTAAGGGTTCTATCATTGTACAATCGAAGTATACTTAAG

Geisha GTGTTACCCAGATTCAAGTCCTTAAGGGTTCTATCATTGTACAATCGAAGTATACTTAAG

************************************************************

Et039 TTGCCTGACTCGTTTAGGCATTT**CA**AGCAACT**T**CG**A**ATCCTGAATCTGTCTC**A**TAC**A**C**C**C

Caturra TTGCCTGACTCGTTTAGGCATTT**AC**AGCAACT**A**CG**T**ATCCTGAATCTGTCTC**G**TAC**G**C**A**C

Geisha TTGCCTGACTCGTTTAGGCATTT**AC**AGCAACT**A**CG**T**ATCCTGAATCTGTCTC**G**TAC**G**C**A**C

*********************** ******* ** **************** *** * *

Et039 ATAGAAAAGCTACCGGACTGGATATGTACCTTGTACAATCTACAAACTTTGTTGTTGTCA

Caturra ATAGAAAAGCTACCGGACTGGATATGTACCTTGTACAATCTACAAACTTTGTTGTTGTCA

Geisha ATAGAAAAGCTACCGGACTGGATATGTACCTTGTACAATCTACAAACTTTGTTGTTGTCA

************************************************************

Et039 GATTGCAAACACCT**T**GAGGAGTTGCCCAAAGATCTAGGAAAGCTAATTAATTTGTGTTTT

Caturra GATTGCAAACACCT**C**GAGGAGTTGCCCAAAGATCTAGGAAAGCTAATTAATTTGTGTTTT

Geisha GATTGCAAACACCT**C**GAGGAGTTGCCCAAAGATCTAGGAAAGCTAATTAATTTGTGTTTT

************** *********************************************

Et039 CTAGATATTAGTGGGGTCCCGCTGAAGAAAATGCCAATGAAAATGGGTAGACTGAAAAAC

Caturra CTAGATATTAGTGGGGTCCCGCTGAAGAAAATGCCAATGAAAATGGGTAGACTGAAAAAC

Geisha CTAGATATTAGTGGGGTCCCGCTGAAGAAAATGCCAATGAAAATGGGTAGACTGAAAAAC

************************************************************

Et039 CTTCAAGTTTTAACT**G**CTTTTGTGGCAGGCAAGGACTATGGTTTGACAATTGAGGAGTTG

Caturra CTTCAAGTTTTAACT**A**CTTTTGTGGCAGGCAAGGACTATGGTTTGACAATTGAGGAGTTG

Geisha CTTCAAGTTTTAACT**A**CTTTTGTGGCAGGCAAGGACTATGGTTTGACAATTGAGGAGTTG

*************** ********************************************

Et039 GGAAAACTTCCTATGCTTGGCGGTAAGCTACT**C**ATTTCTGGGCTGGAAAAA**A**TTTCTGGT

Caturra GGAAAACTTCCTATGCTTGGCGGTAAGCTACT**A**ATTTCTGGGCTGGAAAAA**G**TTTCTGGT

Geisha GGAAAACTTCCTATGCTTGGCGGTAAGCTACT**A**ATTTCTGGGCTGGAAAAA**G**TTTCTGGT

******************************** ****************** ********

Et039 GGAAGAGAAGCATCAATGGCAAACATAAAGGGCAAGAA**C**CAGCTTGAAAGTTTAACTTTG

Caturra GGAAGAGAAGCATCAATGGCAAACATAAAGGGCAAGAA**A**CAGCTTGAAAGTTTAACTTTG

Geisha GGAAGAGAAGCATCAATGGCAAACATAAAGGGCAAGAA**A**CAGCTTGAAAGTTTAACTTTG

************************************** *********************

Et039 AAGTGGAATGATGATGGTAATGGTTCGCAAGTTGCCAGAGATGTGCTTGATGGTCTACAG

Caturra AAGTGGAATGATGATGGTAATGGTTCGCAAGTTGCCAGAGATGTGCTTGATGGTCTACAG

Geisha AAGTGGAATGATGATGGTAATGGTTCGCAAGTTGCCAGAGATGTGCTTGATGGTCTACAG

************************************************************

Et039 CCTCATTCAAGTATAAAACATTTGAAAATCAATGGATATTGTGGGACAAGATTTCCTAAC

Caturra CCTCATTCAAGTATAAAACATTTGAAAATCAATGGATATTGTGGGACAAGATTTCCTAAC

Geisha CCTCATTCAAGTATAAAACATTTGAAAATCAATGGATATTGTGGGACAAGATTTCCTAAC

************************************************************

Et039 TGGCT**C**GAAACCCCTTCATTTTGCCATATAGAATCCATAAGTCTGAT**G**AATTGTGAATAT

Caturra TGGCT**G**GAAACCCCTTCATTTTGCCATATAGAATCCATAAGTCTGAT**C**AATTGTGAATAT

Geisha TGGCT**G**GAAACCCCTTCATTTTGCCATATAGAATCCATAAGTCTGAT**C**AATTGTGAATAT

***** ***************************************** ************

Et039 TGCTTA**A**GCTTGCCCGCACTTGGGCAGCTTCAGTCCTTGAAATCCCTTGAAATTGTTGGA

Caturra TGCTTA**C**GCTTGCCCGCACTTGGGCAGCTTCAGTCCTTGAAATCCCTTGAAATTGTTGGA

Geisha TGCTTA**C**GCTTGCCCGCACTTGGGCAGCTTCAGTCCTTGAAATCCCTTGAAATTGTTGGA

****** *****************************************************

Et039 ATGAGTAACAT**A**TCAGCCTTGACTGAAGACATGTATTATGGGGACAATTGTGAAATTAAA

Caturra ATGAGTAACAT**G**TCAGCCTTGACTGAAGACATGTATTATGGGGACAATTGTGAAATTAAA

Geisha ATGAGTAACAT**G**TCAGCCTTGACTGAAGACATGTATTATGGGGACAATTGTGAAATTAAA

*********** ************************************************

Et039 CCTTTTCCATCTCTCAGAA**T**ATTCAAGATTGAGAATATGCAACAGTTGGAGAAATGGGAT

Caturra CCTTTTCCATCTCTCAGAA**A**ATTCAAGATTGAGAATATGCAACAGTTGGAGAAATGGGAT

Geisha CCTTTTCCATCTCTCAGAA**A**ATTCAAGATTGAGAATATGCAACAGTTGGAGAAATGGGAT

******************* ****************************************

Et039 **A**TACCAGAAGGTGAAGTCTTTTGCAGTCTTGAAAACCTCTCTATAAT**G**GATTG**T**CCCAAA

Caturra **G**TACCAGAAGGTGAAGTCTTTTGCAGTCTTGAAAACCTCTCTATAAT**T**GATTG**C**CCCAAA

Geisha **G**TACCAGAAGGTGAAGTCTTTTGCAGTCTTGAAAACCTCTCTATAAT**T**GATTG**C**CCCAAA

********************************************** ***** ******

Et039 CTCGT**C**GGAGAATTGCCCAAACAAC**T**TTCATCACTC**A**AAATACTGGAGATATCTGGGTGT

Caturra CTCGT**T**GGAGAATTGCCCAAACAAC**G**TTCATCACTC**G**AAATACTGGAGATATCTGGGTGT

Geisha CTCGT**T**GGAGAATTGCCCAAACAAC**G**TTCATCACTC**G**AAATACTGGAGATATCTGGGTGT

***** ******************* ********** ***********************

Et039 GACAGGTTTGTGCTCAGTAATGGTCGATTGAGCATCTTGGA**G**GAACACATTCAACAACTC

Caturra GACAGGTTTGTGCTCAGTAATGGTCGATTGAGCATCTTGGA**C**GAACACATTCAACAACTC

Geisha GACAGGTTTGTGCTCAGTAATGGTCGATTGAGCATCTTGGA**C**GAACACATTCAACAACTC

***************************************** ******************

Et039 TCATCTCTTCGTCAATTGACTGTTTCACGCATGGAGAATTTGAAAGAGCTAT**C**CCCAGAA

Caturra TCATCTCTTCGTCAATTGACTGTTTCACGCATGGAGAATTTGAAAGAGCTAT**T**CCCAGAA

Geisha TCATCTCTTCGTCAATTGACTGTTTCACGCATGGAGAATTTGAAAGAGCTAT**T**CCCAGAA

**************************************************** *******

Et039 CTGAACAAGTT**T**GCCTGTCTTGAGTGGTTGAAAATTAGAGATTGTGACTCTATCAAAGTT

Caturra CTGAACAAGTT**A**GCCTGTCTTGAGTGGTTGAAAATTAGAGATTGTGACTCTATCAAAGTT

Geisha CTGAACAAGTT**A**GCCTGTCTTGAGTGGTTGAAAATTAGAGATTGTGACTCTATCAAAGTT

*********** ************************************************

Et039 GTCTCGCTTGGCTTGTTCCCCATGCTAAAA**G**A**T**GTTCACATTGA**T**A**CC**TGCAAGAGTATG

Caturra GTCTCGCTTGGCTTGTTCCCCATGCTAAAA**C**A**C**GTTCACATTGA**A**A**AG**TGCAAGAGTATG

Geisha GTCTCGCTTGGCTTGTTCCCCATGCTAAAA**C**A**C**GTTCACATTGA**A**A**AG**TGCAAGAGTATG

****************************** * *********** * ************

Et039 GAGATGCT**G**TCCGTTCCTCCACGTGGAATTGGGAATCAAAGTAGTA**T**TCTAACTTCACTT

Caturra GAGATGCT**C**TCCGTTCCTCCACGTGGAATTGGGAATCAAAGTAGTA**G**TCTAACTTCACTT

Geisha GAGATGCT**C**TCCGTTCCTCCACGTGGAATTGGGAATCAAAGTAGTA**G**TCTAACTTCACTT

******** ************************************* *************

Et039 CAATCCTTGT**T**GATCTGGGACTGTGATAATCTAATGTCTTTTCCAGATGAGGGATTGCCA

Caturra CAATCCTTGT**G**GATCTGGGACTGTGATAATCTAATGTCTTTTCCAGATGAGGGATTGCCA

Geisha CAATCCTTGT**G**GATCTGGGACTGTGATAATCTAATGTCTTTTCCAGATGAGGGATTGCCA

********** *************************************************

Et039 GCTCCCAACTTA**A**AGTCGATG**CG**CATCG**GGC**GTTGCAAGAAGCTCAAGTCACTGCCGGCA

Caturra GCTCCCAACTTA**G**AGTCGATG**AA**CATCG**CAT**GTTGCAAGAAGCTCAAGTCACTGCCGGCA

Geisha GCTCCCAACTTA**G**AGTCGATG**AA**CATCG**CAT**GTTGCAAGAAGCTCAAGTCACTGCCGGCA

************ ******** ***** *****************************

Et039 AGGATGGAA**T**CACTTCTTCCATCTCTT**C**AA**C**GACTCACATTAATC**CG**TTGTCCAGAAATT

Caturra AGGATGGAA**C**CACTTCTTCCATCTCTT**G**AA**G**AACTCACATTAATC**GA**TTGTCCAGAAATT

Geisha AGGATGGAA**C**CACTTCTTCCATCTCTT**G**AA**G**AACTCACATTAATC**GA**TTGTCCAGAAATT

********* ***************** ** ************* *************

Et039 GAGCGCTTTCCAGAGGGGGGTTTGCCCACCAGCCTAC**G**AACACTT**T**GGATTACCT**C**TTGT

Caturra GAGCGCTTTCCAGAGGGGGGTTTGCCCACCAGCCTAC**A**AACACTT**C**GGATTACCT**T**TTGT

Geisha GAGCGCTTTCCAGAGGGGGGTTTGCCCACCAGCCTAC**A**AACACTT**C**GGATTACCT**T**TTGT

************************************* ******* ********* ****

Et039 **A**AAAAGCTCC**T**GAC**G**AGTCCAAGAGAGTGGGATTTGATGAGACTCCCCTGCCTCCGATCC

Caturra **G**AAAAGCTCC**C**GAC**A**AGTCCAAGAGAGTGGGATTTGATGAGACTCCCCTGCCTCCGATCC

Geisha **G**AAAAGCTCC**C**GAC**A**AGTCCAAGAGAGTGGGATTTGATGAGACTCCCCTGCCTCCGATCC

********* *** *********************************************

Et039 TTG**TC**CGTTCATGTCATGGATGAAGCAATAGAGTCCTTTCCAAA**C**GAGGACTGGCTGCTG

Caturra TTG**GG**CGTTCATGTCATGGATGAAGCAATAGAGTCCTTTCCAAA**G**GAGGACTGGCTGCTG

Geisha TTG**GG**CGTTCATGTCATGGATGAAGCAATAGAGTCCTTTCCAAA**G**GAGGACTGGCTGCTG

*** *************************************** ***************

Et039 CCTTGCACTCTTGAAGATCTC**A**AACTCTTCC**T**A**A**GT**G**AGAATATCAAAAC**A**CTAAA**C**TAT

Caturra CCTTGCACTCTTGAAGATCTC**G**AACTCTTCC**G**A**G**GT**A**AGAATATCAAAAC**G**CTAAA**T**TAT

Geisha CCTTGCACTCTTGAAGATCTC**G**AACTCTTCC**G**A**G**GT**A**AGAATATCAAAAC**G**CTAAA**T**TAT

********************* ********* * ** ************* ***** ***

Et039 TCGGGT**C**TTCA**G**CACCTCACCTCTCTTCAAA**G**TCTA**GT**AATC**AC**T**GA**GTGCAGTCTCCTC

Caturra TCGGGT**T**TTCA**A**CACCTCACCTCTCTTCAAA**A**TCTA**CG**AATC**GG**T**CG**GTGCAGTCTCCTC

Geisha TCGGGT**T**TTCA**A**CACCTCACCTCTCTTCAAA**A**TCTA**CG**AATC**GG**T**CG**GTGCAGTCTCCTC

****** **** ******************* **** **** * *************

Et039 CAGT**G**GCT**G**CCAGAAGAGGGACTGCCTGCCTCCCTCACTAAACTGGAAATCAGGGGCTGT

Caturra CAGT**C**GCT**C**CCAGAAGAGGGACTGCCTGCCTCCCTCACTAAACTGGAAATCAGGGGCTGT

Geisha CAGT**C**GCT**C**CCAGAAGAGGGACTGCCTGCCTCCCTCACTAAACTGGAAATCAGGGGCTGT

**** *** ***************************************************

Et039 CCACTGCTGAAACCAAGGTTAGAATGGGAGAAAGGACGAGACTGGTCCAAGGTTGCCCAC

Caturra CCACTGCTGA--------------------------------------------------

Geisha CCACTGCTGAAACCAAGGTTAGAATGGGAGAAAGGACGAGACTGGTCCAAGGTTGCCCAC

**********

Et039 ATCCCCTGCATAATATATATCATTTCTGGGTTTGAATGTTATGGACCATTCACTGCTATC

Caturra ------------------------------------------------------------

Geisha ATCCCCTGCATA------------------------------------------------

Et039 TCGCCCTCAGTTATTGCAGGAAGAGGAAAGAGAAGATTGGTTGAAGATAGTTCGTACTTC

Caturra ------------------------------------------------------------

Geisha ------------------------------------------------------------

Et039 GGGGATGTTAGCGATGAACTGAAGCATTCAAATGTGATTCTTTTGGACAAGATTGAAATC

Caturra ------------------------------------------------------------

Geisha ------------------------------------------------------------

Et039 TAG

Caturra ---

Geisha ---
