# Supplementary material for: Genome-wide association study for resistance to Pseudomonas syringae pv. garcae in Coffea arabica
Source: Front Plant Sci. 2022 Oct 18;13:989847. doi: 10.3389/fpls.2022.989847 (PMC9624508; doi:10.3389/fpls.2022.989847)
Supplement: Supplementary Figure 1 — Histogram of the disease distribution, values of response to Bacterial Halo Blight obtained in field evaluation (Mohan et al., 1978; Ito et al., 2008). The X-axis represents the classes of distribution for the 120 C. arabica wild accessions (blue), 11 C. arabica cultivars (red) and BA-10 genotype evaluated. The Y-axis shows the count of C. arabica genotypes in each category. [file DataSheet_1.zip › Supplementary Material 2.DOCX]

**Supplementary Material 2.** The protein sequences multiple alignment. The protein sequences of 1,137 amino acids correspond to the genomic regions of Et039, Caturra and Geisha used to the SNPs identification. Changes in amino acid sequence correspond to nsSNPs.

Et039 KLKNNLLAVGAVLDDAENKEISNQAVKEWLVELHEIVYQADDLLDEINTEVLRVQVESEY 60

Caturra KLKINLLAVGAVLDDAENKEISSQAVKEWLEEIHEIVYQADDLLDEINTEALRVKVESEY 60

Geisha KLKINLLAVGAVLDDAENKEISSQAVKEWLEEIHEIVYQADDLLDEINTEALRVKVESEY 60

*** ******************.******* *:*****************.***:*****

Et039 QSSTNILASASTYFSSFSNQFFKRIMPDIEKVVISLEGFIQQINPLGLQVVEPKIRSYRL 120

Caturra KSSTSFLVSASTYISSFSNQFFKRIMPEIEKVVISLEGFIQQINPLGLQVVEPKIRSYRL 120

Geisha KSSTSFLVSASTYISSFSNQFFKRIMPEIEKVVISLEGFIQQINPLGLQVVEPKIRSYRL 120

:***.:*.*****:*************:********************************

Et039 PSTSLVDEDSVYGRDIDKEKIFQLLLSEDNRGDDIAVVPIVGQGGIGKTTLVQLVYNDKR 180

Caturra PSTSLVDEDAVYGRDVEEENIIQMLLSEDEKGDNVTVVSIVGQGGIGKTTLAQLVYNDKR 180

Geisha PSTSLVDEDAVYGRDVEEENIIQMLLSEDEKGDNVTVVSIVGQGGIGKTTLAQLVYNDKR 180

*********:*****:::*:*:*:*****::**:::** ************.********

Et039 VKNYFPTKAWVCVSEEYDATRITKELLRELGISFSDSSESLNSLQVKLQQGLTDKKFLLV 240

Caturra VKNHFPTKAWVCVSEEYNATRITKELLREFDISFSDSGESLNSLQVKLQQGLTDKKFLLV 240

Geisha VKNHFPTKAWVCVSEEYNATRITKELLREFDISFSDSGESLNSLQVKLQQGLTDKKFLLV 240

***:*************:***********:.******.**********************

Et039 LDDVWNDDYDDWDKLKMLVKGGSEGSKIIVTTRDERIALMMSRKMSIHYLDLLSEEDSWV 300

Caturra LDDVWNDDYDDWYKLKMLVKGGSEGSKIIVTTRDERIALMMGHKMSIHHLGLLSEEDSWV 300

Geisha LDDVWNDDYDDWYKLKMLVKGGSEGSKIIVTTRDERIALMMGHKMSIHHLGLLSEEDSWV 300

************ ****************************.:*****:*.*********

Et039 LFEKHAFGCKDNEIRPELEVIGKKIVNKCEGLPLAVKTIAGLLRSRSTVEEWEEILRNDL 360

Caturra LFEKHAFGGKDNEIRPELEVIGKKIVNKCEGLPLAVKTIAGLLRSRSTVEEWEEILRNDL 360

Geisha LFEKHAFGGKDNEIRPELEVIGKKIVNKCEGLPLAVKTIAGLLRSRSTVEEWEEILRNDL 360

******** ***************************************************

Et039 WNQTRNPNGILPALRLSYMHLPSHLKRCFAYCAVFHKDFWFSKQEIIQLWHANGLLEHPR 420

Caturra WNQTRNPNGILPALRLSYMHLPSHLKRCFACCAVFHKDFWFSKQEIIQLWHANGLLEHPR 420

Geisha WNQTRNPNGILPALRLSYMHLPSHLKRCFACCAVFHKDFWFSKQEIIQLWHANGLLEHPR 420

****************************** *****************************

Et039 NNESIEDIGGVYLRELRLRSLLWQSIDNTFSMHDLINDLARFVSGKYCLRLEDHYPGYGT 480

Caturra NNESIEDIGGVYLRELRLRSLLWQSTNNTFSMHDLINDLARFVSGKYCLRLEDHYPGYGT 480

Geisha NNESIEDIGGVYLRELRLRSLLWQSTNNTFSMHDLINDLARFVSGKYCLRLEDHYPGYGT 480

************************* :*********************************

Et039 TASVRNFTYYPSMYDTFDKLKLLREAKSLRTFYPVCRSNFAFGDEIISNKFLHDVLPRFK 540

Caturra TASVRNFTYYPSKYDTFDKLKLLREAKSLRTFYPVCRSNFAFSGEIISNKFLHDVLPRFK 540

Geisha TASVRNFTYYPSKYDTFDKLKLLREAKSLRTFYPVCRSNFAFSGEIISNKFLHDVLPRFK 540

************ *****************************..****************

Et039 SLRVLSLYNRSILKLPDSFRHFKQLRILNLSHTPIEKLPDWICTLYNLQTLLLSDCKHLE 600

Caturra SLRVLSLYNRSILKLPDSFRHLQQLRILNLSRTHIEKLPDWICTLYNLQTLLLSDCKHLE 600

Geisha SLRVLSLYNRSILKLPDSFRHLQQLRILNLSRTHIEKLPDWICTLYNLQTLLLSDCKHLE 600

*********************::********:* **************************

Et039 ELPKDLGKLINLCFLDISGVPLKKMPMKMGRLKNLQVLTAFVAGKDYGLTIEELGKLPML 660

Caturra ELPKDLGKLINLCFLDISGVPLKKMPMKMGRLKNLQVLTTFVAGKDYGLTIEELGKLPML 660

Geisha ELPKDLGKLINLCFLDISGVPLKKMPMKMGRLKNLQVLTTFVAGKDYGLTIEELGKLPML 660

***************************************:********************

Et039 GGKLLISGLEKISGGREASMANIKGKNQLESLTLKWNDDGNGSQVARDVLDGLQPHSSIK 720

Caturra GGKLLISGLEKVSGGREASMANIKGKKQLESLTLKWNDDGNGSQVARDVLDGLQPHSSIK 720

Geisha GGKLLISGLEKVSGGREASMANIKGKKQLESLTLKWNDDGNGSQVARDVLDGLQPHSSIK 720

***********:**************:*********************************

Et039 HLKINGYCGTRFPNWLETPSFCHIESISLMNCEYCLSLPALGQLQSLKSLEIVGMSNISA 780

Caturra HLKINGYCGTRFPNWLETPSFCHIESISLINCEYCLRLPALGQLQSLKSLEIVGMSNMSA 780

Geisha HLKINGYCGTRFPNWLETPSFCHIESISLINCEYCLRLPALGQLQSLKSLEIVGMSNMSA 780

*****************************:****** ********************:**

Et039 LTEDMYYGDNCEIKPFPSLRIFKIENMQQLEKWDIPEGEVFCSLENLSIMDCPKLVGELP 840

Caturra LTEDMYYGDNCEIKPFPSLRKFKIENMQQLEKWDVPEGEVFCSLENLSIIDCPKLVGELP 840

Geisha LTEDMYYGDNCEIKPFPSLRKFKIENMQQLEKWDVPEGEVFCSLENLSIIDCPKLVGELP 840

******************** *************:**************:**********

Et039 KQLSSLKILEISGCDRFVLSNGRLSILEEHIQQLSSLRQLTVSRMENLKELSPELNKFAC 900

Caturra KQRSSLEILEISGCDRFVLSNGRLSILDEHIQQLSSLRQLTVSRMENLKELFPELNKLAC 900

Geisha KQRSSLEILEISGCDRFVLSNGRLSILDEHIQQLSSLRQLTVSRMENLKELFPELNKLAC 900

** ***:********************:*********************** *****:**

Et039 LEWLKIRDCDSIKVVSLGLFPMLKDVHIDTCKSMEMLSVPPRGIGNQSSILTSLQSLLIW 960

Caturra LEWLKIRDCDSIKVVSLGLFPMLKHVHIEKCKSMEMLSVPPRGIGNQSSSLTSLQSLWIW 960

Geisha LEWLKIRDCDSIKVVSLGLFPMLKHVHIEKCKSMEMLSVPPRGIGNQSSSLTSLQSLWIW 960

************************.***:.******************* ******* **

Et039 DCDNLMSFPDEGLPAPNLKSMRIGRCKKLKSLPARMESLLPSLQRLTLIRCPEIERFPEG 1020

Caturra DCDNLMSFPDEGLPAPNLESMNIACCKKLKSLPARMEPLLPSLEELTLIDCPEIERFPEG 1020

Geisha DCDNLMSFPDEGLPAPNLESMNIACCKKLKSLPARMEPLLPSLEELTLIDCPEIERFPEG 1020

******************:**.*. ************ *****:.**** **********

Et039 GLPTSLRTLWITSCKKLLTSPREWDLMRLPCLRSLSVHVMDEAIESFPNEDWLLPCTLED 1080

Caturra GLPTSLQTLRITFCEKLPTSPREWDLMRLPCLRSLGVHVMDEAIESFPKEDWLLPCTLED 1080

Geisha GLPTSLQTLRITFCEKLPTSPREWDLMRLPCLRSLGVHVMDEAIESFPKEDWLLPCTLED 1080

******:** ** *:** *****************.************:***********

Et039 LKLFLSENIKTLNYSGLQHLTSLQSLVITECSLLQWLPEEGLPASLTKLEIRGCPLL 1137

Caturra LELFRGKNIKTLNYSGFQHLTSLQNLRIGRCSLLQSLPEEGLPASLTKLEIRGCPLL 1137

Geisha LELFRGKNIKTLNYSGFQHLTSLQNLRIGRCSLLQSLPEEGLPASLTKLEIRGCPLL 1137

*:** .:*********:*******.* * .***** *********************
