# Supplementary material for: Genome-wide association study for resistance to Pseudomonas syringae pv. garcae in Coffea arabica
Source: Front Plant Sci. 2022 Oct 18;13:989847. doi: 10.3389/fpls.2022.989847 (PMC9624508; doi:10.3389/fpls.2022.989847)
Supplement: Supplementary Figure 1 — Histogram of the disease distribution, values of response to Bacterial Halo Blight obtained in field evaluation (Mohan et al., 1978; Ito et al., 2008). The X-axis represents the classes of distribution for the 120 C. arabica wild accessions (blue), 11 C. arabica cultivars (red) and BA-10 genotype evaluated. The Y-axis shows the count of C. arabica genotypes in each category. [file DataSheet_1.zip › Supplementary Table 1.DOCX]

**Supplementary Table 1.** List of the 132 accessions of *C. arabica* and its respective cluster, according to the PCA divided into 3 clusters. The characteristics of the 120 Ethiopian accessions of *C. arabica* are described according to the FAO registry (FAO, 1968). NI = not informed.

| **Accessions** | **Great Rift Valley side** | **Location, Province** | **Coordinates** | **Accession characterization** | **cluster** |  |
| --- | --- | --- | --- | --- | --- | --- |
|  |  |  |  |  |  |  |
| Bourbon | - | - | - | - | green |  |
| CatuaiV26 | - | - | - | - | green |  |
| IAPAR59 | - | - | - | - | green |  |
| IPR100 | - | - | - | - | green |  |
| IPR101 | - | - | - | - | green |  |
| IPR102 | - | - | - | - | green |  |
| IPR103 | - | - | - | - | green |  |
| IPR104 | - | - | - | - | green |  |
| IPR105 | - | - | - | - | green |  |
| IPR107 | - | - | - | - | green |  |
| IPR99 | - | - | - | - | green |  |
| E021_011 | EAST | Road to Soddu, Sidamo Province | 7°8N 37°58E | Farm open field | green |  |
| E261_052 | WEST | Doyo, Kaffa Jimma Province | 7°37N 36°46E | Farm open field | green |  |
| E012_136 | EAST | 5 km northwest of Harar, Harar Province | 9°19N 42°6 E | Farm open field | red |  |
| E016_298 | EAST | Southeast of Shashamanna, Shoa Province | 7°7N 38°40E | Farm open field | red |  |
| E017_419 | EAST | Wanago, Sidamo Province | 6°20N 38°15E | Wet processed | red |  |
| E018_494 | EAST | Yirga Cheffe, Sidamo Province | 6°12N 38°13E | Domesticated Shadow | red |  |
| E022_163 | EAST | Soddu, Sidamo Province | 6°52N 37°46E | Farm open field | red |  |
| E025_308 | WEST | 5 km South of Welkite, Shoa Province | NI | NI | red |  |
| E030_075 | WEST | Doyo, Kaffa Jimma Province | 7°37N 36°46E | Farm open field | red |  |
| E037_676 | EAST | Wondo Genet, Shoa Province | 7°7N 38°40E | Farm open field | red |  |
| E038_043 | WEST | Bada Buna, Kaffa Jimma Province | 7°40N 38°52E | Naturalized | red |  |
| E039_434 | WEST | Chochi, Kaffa Jimma Province | 7°5N 36°45E | Wet processed | red |  |
| E044_122 | WEST | Headquarters north of Jimma, Kaffa Jimma Province | 7°57N 36°48E | Naturalized Forest | red |  |
| E046_021 | WEST | Headquarters north of Jimma, Kaffa Jimma Province | 7°57N 36°48E | Naturalized Forest | red |  |
| E047_267 | WEST | Headquarters north of Jimma, Kaffa Jimma Province | 7°57N 36°48E | Naturalizado Forest | red |  |
| E055_005 | WEST | Bada Buna, Kaffa Jimma Province | 7°4N 38°52E | Naturalized | red |  |
| E057_496 | WEST | Bada Buna, Kaffa Jimma Province | 7°4N 38°52E | Naturalized | red |  |
| E061_126 | WEST | Bada Buna, Kaffa Jimma Province | 7°4N 38°52E | Naturalized | red |  |
| E068_014 | WEST | Saredo, Kaffa Jimma Province | 8°05N 36°53E | Domesticated Shadow | red |  |
| E071_258 | WEST | Headquarters north of Jimma, Kaffa Jimma Province | 7°57N 36°48E | Naturalized Forest | red |  |
| E080_584 | WEST | Cossa Kabenna, Kaffa Jimma Province | 7°58 N 36°53E | Domesticated | red |  |
| E081_041 | WEST | North of Ghembi, Kaffa Jimma Province | 8°5N 36°54E | Naturalized | red |  |
| E085_085 | WEST | Road from Gera to Afallo, Illubador Province | 7°45N 36°18E | Farm open field | red |  |
| E087_194 | WEST | Edge Gera Village, Kaffa Jimma Province | 7°14N 36°24E | Farm open field | red |  |
| E089_391 | WEST | Between Agaro and Ghera, Kaffa Jimma Province | 7°50N 36°30E | Farm open field | red |  |
| E123a_231 | WEST | Beru, Kaffa Jimma Province | 6°15N 35°14 | Forest | red |  |
| E123b_121 | WEST | Beru, Kaffa Jimma Province | 6°15N 35°14 | Forest | red |  |
| E126_359 | WEST | Gorei, Kaffa Jimma Province | 6°42N 35°34E | NI | red |  |
| E130_169 | WEST | Ota, Kaffa Jimma Province | 7°51N 36°37E | Domesticated | red |  |
| E131_018 | WEST | Ota, Kaffa Jimma Province | 7°51N 36°37E | Domesticated | red |  |
| E146_012 | WEST | Ghera, Illubador Province | 7°14N 36°24E | Farm open field | red |  |
| E148_254 | WEST | Ghera, Illubador Province | 7°14N 36°24E | Farm open field | red |  |
| E152_017 | WEST | Ghera, Illubador Province | 7°14N 36°24E | Farm open field | red |  |
| E159_180 | WEST | Ghera, Illubador Province | 7°14N 36°24E | Farm open field | red |  |
| E164_417 | WEST | Cossa, Kaffa Jimma Province | 7°52N 36°52E | Domesticated | red |  |
| E174_164 | WEST | Suntu Southwest, Kaffa Jimma Province | 8°8N 36°53E | Domesticated | red |  |
| E179_650 | WEST | Between Agaro and Ghera, Kaffa Jimma Province | NI | Mix | red |  |
| E180_070 | WEST | Between Agaro and Ghera, Kaffa Jimma Province | NI | Mix | red |  |
| E183_138 | WEST | Between Agaro and Ghera, Kaffa Jimma Province | NI | Mix | red |  |
| E213_211 | WEST | Cerca de Teppi, Illubabor Province | 7°9N 35°18E | Forest Mix | red |  |
| E221_214 | WEST | Near Teppi, Illubabor Province | 7°9N 35°18E | Forest Mix | red |  |
| E237_071 | EAST | Between Yirgalem and Bira, Sidamo Province | NI | NI | red |  |
| E254_284 | WEST | Chochi, Kaffa Jimma Province | 7°5N 36°45E | NI | red |  |
| E265_101 | WEST | Balt, Kaffa Jimma Province | NI | Shadow | red |  |
| E267_090 | WEST | Balt, Kaffa Jimma Province | NI | Shadow | red |  |
| E270_044 | WEST | Kursi in Chersi, Kaffa Jimma Province | 6°10N 30°33 E 6.179050 | Shadow | red |  |
| E272_143 | WEST | Kolu, Kaffa Jimma Province | 6°95N 35°18E | NI | red |  |
| E279_618 | WEST | 22 km West of Bonga, Kaffa Province | Wush wush | Domesticated | red |  |
| E283_096 | WEST | Wush Wush, Kaffa Jimma Province | Wush wush | Domesticated | red |  |
| E287_029 | WEST | Shebe, Kaffa Jimma Province | Shebe | NI | red |  |
| E298_382 | WEST | Northern Ghembi, Kaffa Jimma Province | 8°5N 36°54 | Naturalized | red |  |
| E301_111 | WEST | Doyo, Kaffa Jimma Province | 7°37N 36°46E | Farm open field | red |  |
| E308_049 | WEST | Doyo, Kaffa Jimma Province | 7°37N 36°46E | Farm open field | red |  |
| E315_081 | WEST | Saredo, Kaffa Jimma Province | 8°05N 36°53E | Domesticated Shadow | red |  |
| E320_145 | WEST | Saredo, Kaffa Jimma Province | 8°05N 36°53E | Domesticated Shadow | red |  |
| E324_093 | WEST | Suntu, Kaffa Jimma Province | Suntu | NI | red |  |
| E326_124 | WEST | Ghera, Kaffa Jimma Province | 7°14N 36°24E | Farm open field | red |  |
| E327_032 | WEST | Ghera, Kaffa Jimma Province | 7°14N 36°24E | Farm open field | red |  |
| E332_023 | WEST | Ghera, Kaffa Jimma Province | 7°14N 36°24E | Farm open field | red |  |
| E335_219 | WEST | Ghera, Kaffa Jimma Province | 7°14N 36°24E | Farm open field | red |  |
| E338_218 | WEST | Ghera, Kaffa Jimma Province | 7°14N 36°24E | Farm open field | red |  |
| E340_179 | WEST | Ghera, Kaffa Jimma Province | 7°14N 36°24E | Farm open field | red |  |
| E344_008 | WEST | Ghera, Kaffa Jimma Province | 7°14N 36°24E | Farm open field | red |  |
| E351_248 | WEST | Ghera, Kaffa Jimma Province | 7°14N 36°24E | Farm open field | red |  |
| E363_735 | WEST | Trail to Afallo, Illubador | 7°45N 36°18E | NI | red |  |
| E364_059 | WEST | Trail to Afallo, Illubador Province | 7°45N 36°18E | NI | red |  |
| E368_600 | WEST | Trail to Afallo, Illubador | 7°45N 36°18E | NI | red |  |
| E370_196 | WEST | Trail to Afallo, Illubador Province | 7°45N 36°18E | NI | red |  |
| E383_142 | WEST | Trail to Afallo trail, Kaffa Province | 7°43N 36°18E | NI | red |  |
| E386_131 | WEST | 5 km North of Afallo, Kaffa Jimma Province | 7°43N 36°18E | Naturalized | red |  |
| E389_133 | WEST | 5 km north of Afallo, Kaffa Jimma Province | 7°43N 36°18E | Naturalized | red |  |
| E401_643 | WEST | Ota, Kaffa Jimma Province | 7°51N 36°37E | Domesticated | red |  |
| E404_135 | WEST | Ota, Kaffa Jimma Province | 7°51N 36°37E | Domesticated | red |  |
| E408_001 | WEST | Between Bonga and Wush Wush, Kaffa Jimma Province | 7°15N 36°13E | NI | red |  |
| E409_114 | WEST | Between Bonga and Wush Wush, Kaffa Jimma Province | 7°15N 36°13E | NI | red |  |
| E419_098 | WEST | Bonga, Kaffa Jimma Province | 7°15N 36°13E | Cultivated | red |  |
| E464_417 | WEST | 7 km southeast of Teppi, Illubabor Province | NI | Forest | red |  |
| E467_045 | WEST | 10 Km southwest of Agaro, Fichi Village, Kaffa Province | 7°39N 36°33E | Domesticated | red |  |
| E478_408 | WEST | North of Ghembi, Kaffa Jimma Province | 8°5N 36°54E | Naturalized | red |  |
| E481_238 | WEST | North of Ghembi, Kaffa Jimma Province | 8°5N 36°54E | Naturalized | red |  |
| E486_189 | WEST | North of Ghembi, Kaffa Jimma Province | 8°5N 36°54E | Naturalized | red |  |
| E490_516 | WEST | North of Ghembi, Kaffa Jimma Province | 8°5N 36°54E | Naturalized | red |  |
| E494_173 | WEST | Cossa Kabenna, Kaffa Jimma Province | 7°58 N 36°53E | Domesticated | red |  |
| E534_036 | WEST | Between Bonga and Wush Wush, Kaffa Jimma Province | 7°15N 36°12 | Cultivated/ Naturalized | red |  |
| E546_118 | WEST | Plantation in Wush Wush, Kaffa Jimma Province | 7°15N 36°8 | Domesticated | red |  |
| E552_323 | WEST | Plantação em Wush Wush, Kaffa Jimma Province | 7°15N 36°8 | Domesticated | red |  |
| E565_010 | WEST | Zeghie, Gojjam Province | 11°42N 37°20 | Cultivated in Forest | red |  |
| BA10_057 | - | - | - | - | blue |  |
| E041_079 | WEST | Headquarters north of Jimma, Kaffa Jimma Province | 7°57N 36°48E | Naturalized Forest | blue |  |
| E114_447 | WEST | Mizan Tefari, Kaffa Jimma Province | 6°55N 35°25E | Farm open field | blue |  |
| E116_061 | WEST | Ainamba, Kaffa Jimma Province | 6°35N 35°28E | Naturalized Shadow | blue |  |
| E118_213 | WEST | Korcha, Illubabor Province | 7°8N 35°24E | Forest Tropical | blue |  |
| E124_245 | WEST | Geisha, Kaffa Jimma Province | 6°38N 335°30E | Forest | blue |  |
| E189_119 | WEST | Near Teppi, Illubabor Province | 7°9N 35°18E | Forest Mix | blue |  |
| E190_013 | WEST | Near Teppi, Illubabor Province | 7°9N 35°18E | Forest Mix | blue |  |
| E196_117 | WEST | Near Teppi, Illubabor Province | 7°9N 35°18E | Forest Mix | blue |  |
| E199_011 | WEST | Near Teppi, Illubabor Province | 7°9N 35°18E | Forest Mix | blue |  |
| E201_134 | WEST | Near Teppi, Illubabor Province | 7°9N 35°18E | Forest Mix | blue |  |
| E208_193 | WEST | Near Teppi, Illubabor Province | 7°9N 35°18E | Forest Mix | blue |  |
| E209_031 | WEST | Near Teppi, Illubabor Province | 7°9N 35°18E | Forest Mix | blue |  |
| E218_581 | WEST | Near Teppi, Illubabor Province | 7°9N 35°18E | Forest Mix | blue |  |
| E220_127 | WEST | Near Teppi, Illubabor Province | 7°9N 35°18E | Forest Mix | blue |  |
| E233_015 | WEST | Mizan Tefari Airport, Kaffa Jimma Province | 6°55N35°25E | NI | blue |  |
| E238_022 | EAST | Aleta Wondo Colla, Sidamo Province | NI | NI | blue |  |
| E268_067 | WEST | Kursi in Chersi, Kaffa Jimma Province | 6°10N 30°33E 6.179050 | Shadow | blue |  |
| E331_280 | WEST | Ghera, Kaffa Jimma Province | 7°14N 36°24E | Farm open field | blue |  |
| E428_109 | WEST | 8 km southwest of Teppi, Illubabor Province | 7°10N 35°18E | Forest | blue |  |
| E439_094 | WEST | 8 km east of Teppi, Illubabor Province | 7°10N 35°18E | Forest | blue |  |
| E442_279 | WEST | 8 km east of Teppi, Illubabor Province | 7°10N 35°18E | Forest | blue |  |
| E450_235 | WEST | 8 km east of Teppi, Illubabor Province | 7°10N 35°18E | Forest | blue |  |
| E454_107 | WEST | 10 km southeast of Teppi, Illubabor Province | 7°9N 35°22E | Forest | blue |  |
| E456_062 | WEST | 10 km southeast of Teppi, Illubabor Province | 7°9N 35°22E | Forest | blue |  |
| E457_477 | WEST | 10 km southeast of Teppi, Illubabor Province | 7°9N 35°22E | Forest | blue |  |
| E458_097 | WEST | 10 km southeast of Teppi, Illubabor Province | 7°9N 35°22E | Forest | blue |  |
| E466_125 | WEST | 7 km southeast of Teppi, Illubabor Province | NI | Forest | blue |  |
| E505_140 | WEST | South of Benessa, Kaffa Jimma Province | 6°50N 35°30E | Cultivated | blue |  |
| E511_157 | WEST | Ainamba, Kaffa Jimma Province | 6°53N 35°28E | Naturalized | blue |  |
| E514_129 | WEST | Ainamba, Kaffa Jimma Province | 6°53N 35°28E | Naturalized | blue |  |
| E516_069 | WEST | Tunteta, Kaffa Jimma Province | 6°52N 35°27E | Cultivated | blue |  |
| E571_072 | WEST | Zeghie, Gojjam Province | 11°42N 37°20 | Cultivated Forest | blue |  |
| E621_139 | WEST | Tunteta, Kaffa Jimma Province | 6°52N 35°27E | Cultivated | blue |  |
