# Supplementary material for: Genome-wide association study for resistance to Pseudomonas syringae pv. garcae in Coffea arabica
Source: Front Plant Sci. 2022 Oct 18;13:989847. doi: 10.3389/fpls.2022.989847 (PMC9624508; doi:10.3389/fpls.2022.989847)
Supplement: Supplementary Figure 1 — Histogram of the disease distribution, values of response to Bacterial Halo Blight obtained in field evaluation (Mohan et al., 1978; Ito et al., 2008). The X-axis represents the classes of distribution for the 120 C. arabica wild accessions (blue), 11 C. arabica cultivars (red) and BA-10 genotype evaluated. The Y-axis shows the count of C. arabica genotypes in each category. [file DataSheet_1.zip › Supplementary Table 2.DOCX]

**Supplementary Table 2.** Distribution of 10.034 SNPs (without the information of 1.256 SNPs on chromosome 0), identified in 132 *C. arabica* accessions, along the 22 *C. arabica* chromosomes.

| **Chr** | **% of SNPs** | **average SNP/Mb** |
| --- | --- | --- |
| Chr_1_sg_C | 4.02 | 11.30 |
| Chr_1_sg_E | 5.16 | 12.15 |
| Chr_2_sg_C | 10.35 | 17.81 |
| Chr_2_sg_E | 9.45 | 14.64 |
| Chr_3_sg_C | 3.38 | 10.95 |
| Chr_3_sg_E | 3.67 | 9.92 |
| Chr_4_sg_C | 4.23 | 13.97 |
| Chr_4_sg_E | 4.70 | 13.94 |
| Chr_5_sg_C | 3.88 | 11.61 |
| Chr_5_sg_E | 3.33 | 8.75 |
| Chr_6_sg_C | 5.94 | 14.58 |
| Chr_6_sg_E | 4.57 | 9.79 |
| Chr_7_sg_C | 4.50 | 13.81 |
| Chr_7_sg_E | 3.74 | 11.02 |
| Chr_8_sg_C | 4.83 | 15.47 |
| Chr_8_sg_E | 5.33 | 14.61 |
| Chr_9_sg_C | 2.14 | 11.46 |
| Chr_9_sg_E | 2.63 | 8.41 |
| Chr_10_sg_C | 3.69 | 13.21 |
| Chr_10_sg_E | 3.93 | 12.62 |
| Chr_11_sg_C | 3.11 | 10.54 |
| Chr_11_sg_E | 3.43 | 10.10 |
| Subgenome C | 50.06 | 13.57 |
| Subgenome E | 49.93 | 11.63 |
| *C. arabica* | 100.00 | 12.53 |
