# Supplementary material for: Genome-wide association study for resistance to Pseudomonas syringae pv. garcae in Coffea arabica
Source: Front Plant Sci. 2022 Oct 18;13:989847. doi: 10.3389/fpls.2022.989847 (PMC9624508; doi:10.3389/fpls.2022.989847)
Supplement: Supplementary Figure 1 — Histogram of the disease distribution, values of response to Bacterial Halo Blight obtained in field evaluation (Mohan et al., 1978; Ito et al., 2008). The X-axis represents the classes of distribution for the 120 C. arabica wild accessions (blue), 11 C. arabica cultivars (red) and BA-10 genotype evaluated. The Y-axis shows the count of C. arabica genotypes in each category. [file DataSheet_1.zip › Supplementary Table 3.DOCX]

**Supplementary Table 3.** QTNs associated with resistance to BHB in *C. arabica* cultivars and Ethiopian accession E287. Information from the GBS genotyping HapMap file. When present, the QTNs are colored blue (positive effect) or red (negative effect). Numbers below the cultivar identification are their score for resistance to BHB.

| **QTN (effect)** | **IPR102** | **E287** | **IPR104** | **IAPAR 59** | **IPR105** | **IPR103** | **IPR99** | **IPR100** | **IPR101** | **IPR107** | **CatuaÍ** | **Bourbon** |
| --- | --- | --- | --- | --- | --- | --- | --- | --- | --- | --- | --- | --- |
|  | **0** | **0** | **1** | **2** | **3** | **3** | **4** | **4** | **4** | **5** | **4** | **5** |
| **Chr_0_435_15461_A(+)** | **AA** | **AA** | **GG** | **GG** | **GG** | **AG** | **GG** | **AG** | **GG** | **AG** | **GG** | **GG** |
| **Chr_0_435_15529_A(+)** | **AA** | **AA** | **GG** | **GG** | **GG** | **AG** | **GG** | **AG** | **GG** | **AG** | **GG** | **GG** |
| **Chr_2_sg_E_32049720_G(+)** | **GG** | **GG** | **CC** | **CC** | **CC** | **CC** | **CC** | **CC** | **CC** | **CC** | **CC** | **CC** |
| **Chr_2_sg_E_32049728_G(+)** | **GG** | **GG** | **AA** | **AA** | **AA** | **AA** | **AA** | **AA** | **AA** | **AA** | **AA** | **AA** |
| **Chr_5_sg_C_29867225_G(-)** | **AA** | **AA** | **AA** | **AA** | **AA** | **AA** | **AA** | **AA** | **AA** | **AG** | **AA** | **AA** |
| **Chr_7_sg_C_1131696_C(+)** | **TT** | **TT** | **TT** | **TT** | **TT** | **TT** | **TT** | **TT** | **TT** | **TT** | **TT** | **TT** |
| **Chr_10_sg_C_439213_G(-)** | **AA** | **AA** | **AA** | **AG** | **AA** | **AA** | **AA** | **AG** | **AG** | **AG** | **AG** | **AA** |
| **Chr_11_sg_C_11719181_C(+)** | **TT** | **CT** | **CT** | **CT** | **TT** | **TT** | **TT** | **TT** | **TT** | **TT** | **TT** | **TT** |
| **Chr_1_sg_C_27081268_C(-)** | **TT** | **TT** | **TT** | **TT** | **TT** | **TT** | **CT** | **TT** | **CT** | **TT** | **TT** | **TT** |
| **Chr_5_sg_E_19112445_C(+)** | **TT** | **TT** | **CT** | **CT** | **TT** | **CT** | **TT** | **TT** | **TT** | **TT** | **TT** | **TT** |
| **Chr_7_sg_E_13418072_C(-)** | **CT** | **CT** | **TT** | **TT** | **TT** | **TT** | **TT** | **CT** | **CT** | **TT** | **CC** | **CT** |
|  |  |  |  |  |  |  |  |  |  |  |  |  |
