# Supplementary material for: Genome-wide association study for resistance to Pseudomonas syringae pv. garcae in Coffea arabica
Source: Front Plant Sci. 2022 Oct 18;13:989847. doi: 10.3389/fpls.2022.989847 (PMC9624508; doi:10.3389/fpls.2022.989847)
Supplement: Supplementary Figure 1 — Histogram of the disease distribution, values of response to Bacterial Halo Blight obtained in field evaluation (Mohan et al., 1978; Ito et al., 2008). The X-axis represents the classes of distribution for the 120 C. arabica wild accessions (blue), 11 C. arabica cultivars (red) and BA-10 genotype evaluated. The Y-axis shows the count of C. arabica genotypes in each category. [file DataSheet_1.zip › Supplementary Table 4.DOCX]

**Supplementary Table 4.** Functional annotation of genes near to QTNs associated with resistance to BHB in *C. arabica* identified in this study. ^1^(+) up or (-) downstream position in bp.

| **QTN** | **Chr** | **QTN position (pb)** | **gene** | **gene position (pb)** | **functional annotation** |
| --- | --- | --- | --- | --- | --- |
| Chr_0_435_15461_A; Chr_0_435_15529_A | SSCAFFOLD_434HRSCAF=435 | 15461 15529 | *g000* | 3371 - 3949 | serine threonine- kinase D6PKL2 |
| Chr_0_435_15461_A; Chr_0_435_15529_A | SSCAFFOLD_434HRSCAF=435 | 15461 15529 | *g004* | 6010 - 9243 | peter Pan |
| Chr_0_435_15461_A; Chr_0_435_15529_A | SSCAFFOLD_434HRSCAF=435 | 15461 15529 | *g003* | 12577 - 15723 | NADH dehydrogenase [ubiquinone] iron-sulfur mitochondrial |
| Chr_0_435_15461_A; Chr_0_435_15529_A | SSCAFFOLD_434HRSCAF=435 | 15461 15529 | *g005* | 27203 - 32576 | pentatricopeptide repeat-containing At4g20740 |
| Chr_1_sg_C_27081268_C | 1 sg C | 27081268 | *g009091* | 27050332 - 27051144 | probable carbohydrate esterase At4g34215 |
| Chr_1_sg_C_27081268_C | 1 sg C | 27081268 | *g090114* | 27068672 - 27069181 | Senescence regulator |
| Chr_1_sg_C_27081268_C | 1 sg C | 27081268 | *g009092* | 27074127 - 27074627 | 60S ribosomal L12 |
| Chr_1_sg_C_27081268_C | 1 sg C | 27081268 | *g090102* | 27145876 - 27146376 | arabinogalactan 1 |
| Chr_5_sg_C_29867225_G | 5 sg C | 29867225 | *g009946* | 29803959 - 29805125 | UBP1-associated 2C |
| Chr_5_sg_C_29867225_G | 5 sg C | 29867225 | *g009949* | 29833526 - 29836580 | F-box FBD LRR-repeat At5g56810 |
| Chr_5_sg_C_29867225_G | 5 sg C | 29867225 | *g009954* | 29932443 - 29933030 | S-norcoclaurine synthase-like |
| Chr_7_sg_C_1131696_C | 7 sg C | 1131696 | *g000386* | 1075600 - 1076928 | unnamed protein produc |
| Chr_7_sg_C_1131696_C | 7 sg C | 1131696 | *g003114* | 1096594 - 1105213 | ABC transporter B family member 19 |
| Chr_10_sg_C_439213_G | 10 sg C | 439213 | *g000155* | 377496 - 381747 | glyceraldehyde-3-phosphate dehydrogenase chloroplastic |
| Chr_10_sg_C_439213_G | 10 sg C | 439213 | *g000156* | 381856 - 387033 | serine threonine- kinase endoribonuclease IRE1a-like |
| Chr_10_sg_C_439213_G | 10 sg C | 439213 | *g001147* | 387798 - 389889 | glucan endo-1\|3-beta-glucosidase 12-like |
| Chr_10_sg_C_439213_G | 10 sg C | 439213 | *g001171* | 389849 - 395185 | glutamate receptor |
| Chr_10_sg_C_439213_G | 10 sg C | 439213 | *g000158* | 398157 - 404353 | probable phosphatase 2C 27 |
| Chr_10_sg_C_439213_G | 10 sg C | 439213 | *g001149* | 407122 - 409052 | F-box kelch-repeat At5g42350-like |
| Chr_10_sg_C_439213_G | 10 sg C | 439213 | *g000186* | 409684 - 415410 | U-box domain-containing 44 |
| Chr_10_sg_C_439213_G | 10 sg C | 439213 | *g000187* | 416685 - 424782 | epidermal growth factor receptor substrate 15-like 1 |
| Chr_10_sg_C_439213_G | 10 sg C | 439213 | *g000159* | 429884 - 442299 | Fanconi anemia group J homolog |
| Chr_10_sg_C_439213_G | 10 sg C | 439213 | *g000188* | 444320 - 446130 | HMG1 |
| Chr_10_sg_C_439213_G | 10 sg C | 439213 | *g000016* | 448416 - 453878 | bromodomain-containing 9-like |
| Chr_10_sg_C_439213_G | 10 sg C | 439213 | *g001152* | 465887 - 478668 | serine threonine- kinase CDL1-like |
| Chr_10_sg_C_439213_G | 10 sg C | 439213 | *g000164* | 494450 - 514214 | Niemann-Pick C1 -like |
| Chr_2_sg_E_32049720_G; Chr_2_sg_E_32049728_G | 2 sg E | 32049720 32049728 | *g000028* | 31983987 - 31984681 | NRT1 PTR FAMILY |
| Chr_2_sg_E_32049720_G; Chr_2_sg_E_32049728_G | 2 sg E | 32049720 32049728 | *g010741* | 32125876 -32129550 | disease resistance RPP13 1 |
| Chr_7_sg_E_13418072_C | 7 sg E | 13418072 | *g00044.6* | 13382589 - 13389091 | S-adenosylmethionine carrier chloroplastic mitochondrial |
| Chr_7_sg_E_13418072_C | 7 sg E | 13418072 | *g004498* | 13390060 - 13425286 | unnamed protein product |
| Chr_7_sg_E_13418072_C | 7 sg E | 13418072 | *g000442* | 13476987 - 13482257 | UDP-galactose transporter 2 |
|  |  |  |  |  |  |
