# Supplementary material for: Genome-wide association study for resistance to Pseudomonas syringae pv. garcae in Coffea arabica
Source: Front Plant Sci. 2022 Oct 18;13:989847. doi: 10.3389/fpls.2022.989847 (PMC9624508; doi:10.3389/fpls.2022.989847)
Supplement: Supplementary Figure 1 — Histogram of the disease distribution, values of response to Bacterial Halo Blight obtained in field evaluation (Mohan et al., 1978; Ito et al., 2008). The X-axis represents the classes of distribution for the 120 C. arabica wild accessions (blue), 11 C. arabica cultivars (red) and BA-10 genotype evaluated. The Y-axis shows the count of C. arabica genotypes in each category. [file DataSheet_1.zip › Supplementary Table 5.DOCX]

**Supplementary Table 5.** Primers sequence for the *g000* (Chr_0_434_435) and *g010741* (Chr_2_sg_E) genes, used in RT-qPCR analyses.

| **gene** | **Functional annotation** | **Forward/Reverse** | **Efficiency** |
| --- | --- | --- | --- |
| *g000* (Chr_0_434_435) | protein serine/threonine kinase activity | CAGCCCCTCAGATTTCCTGAT/ | 102% |
|  |  | TCCCCTAATAAGATCCCTTGCA |  |
| *g010741* (Chr_2_sg_E) | CC-NB-LRR protein | GGCTCGAAACCCCTTCATTT/ AGCAATATTCACAATTCATCAGA | 103% |
|  |  |  |  |
|  |  |  |  |
