# Supplementary figures and images for: Genome-wide association study for resistance to Pseudomonas syringae pv. garcae in Coffea arabica
Source: Front Plant Sci. 2022 Oct 18;13:989847. doi: 10.3389/fpls.2022.989847 (PMC9624508; doi:10.3389/fpls.2022.989847)

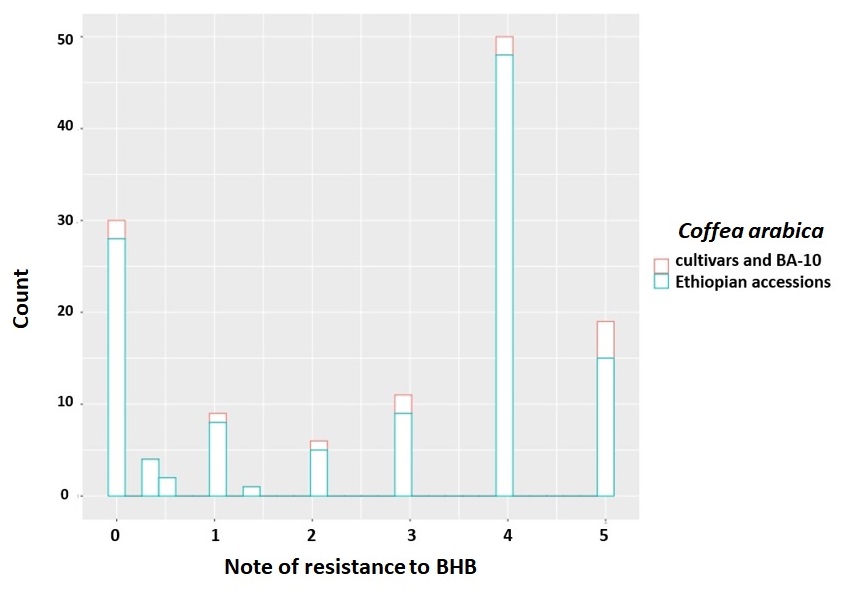

Supplement: Supplementary Figure 1 — Histogram of the disease distribution, values of response to Bacterial Halo Blight obtained in field evaluation (Mohan et al., 1978; Ito et al., 2008). The X-axis represents the classes of distribution for the 120 C. arabica wild accessions (blue), 11 C. arabica cultivars (red) and BA-10 genotype evaluated. The Y-axis shows the count of C. arabica genotypes in each category. [file DataSheet_1.zip › Supplementary Figure 1.JPEG]

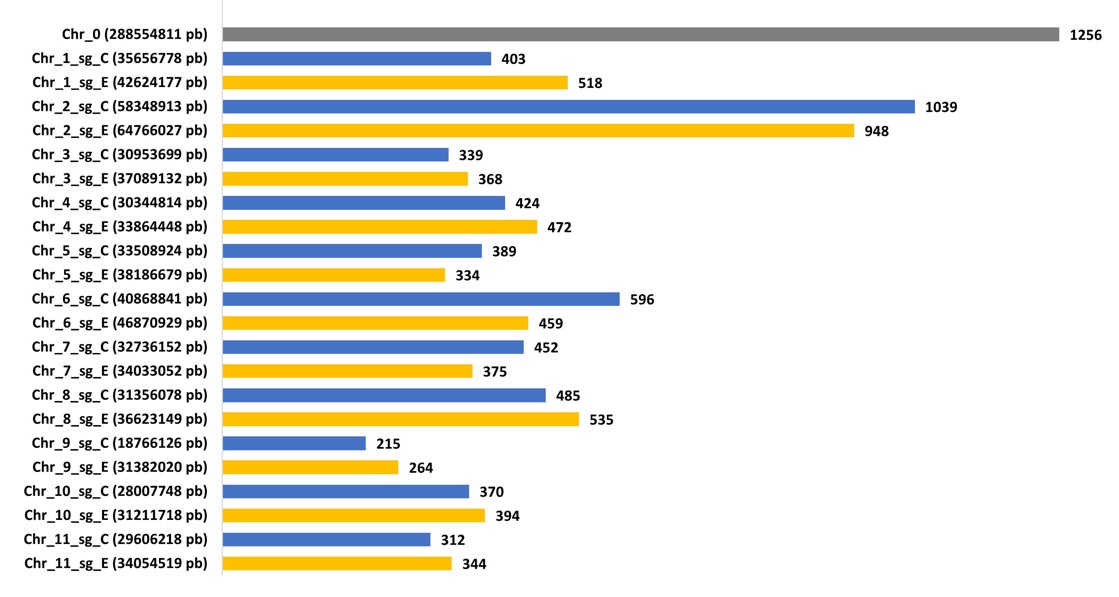

Supplement: Supplementary Figure 1 — Histogram of the disease distribution, values of response to Bacterial Halo Blight obtained in field evaluation (Mohan et al., 1978; Ito et al., 2008). The X-axis represents the classes of distribution for the 120 C. arabica wild accessions (blue), 11 C. arabica cultivars (red) and BA-10 genotype evaluated. The Y-axis shows the count of C. arabica genotypes in each category. [file DataSheet_1.zip › Supplementary Figure 2.JPEG]

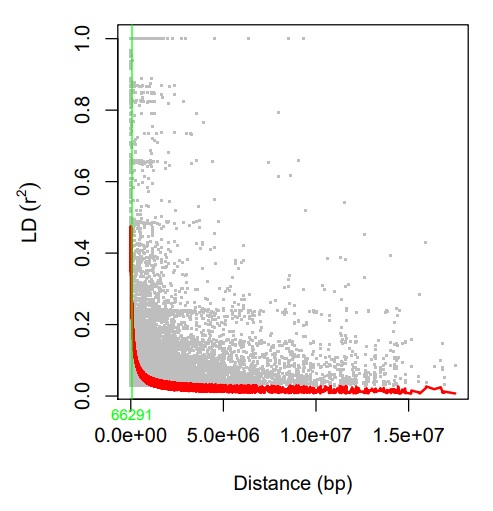

Supplement: Supplementary Figure 1 — Histogram of the disease distribution, values of response to Bacterial Halo Blight obtained in field evaluation (Mohan et al., 1978; Ito et al., 2008). The X-axis represents the classes of distribution for the 120 C. arabica wild accessions (blue), 11 C. arabica cultivars (red) and BA-10 genotype evaluated. The Y-axis shows the count of C. arabica genotypes in each category. [file DataSheet_1.zip › Supplementary Figure 3.JPEG]

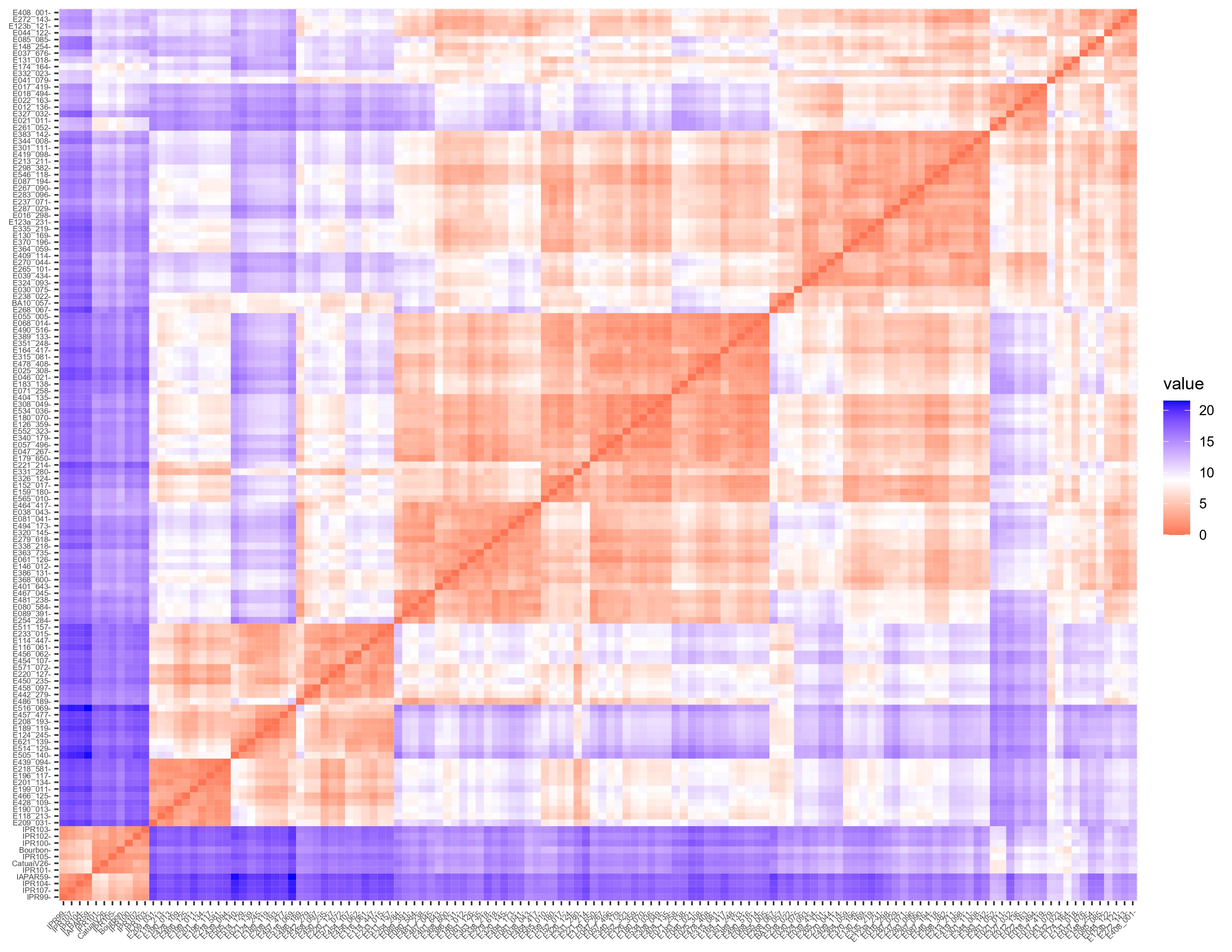

Supplement: Supplementary Figure 1 — Histogram of the disease distribution, values of response to Bacterial Halo Blight obtained in field evaluation (Mohan et al., 1978; Ito et al., 2008). The X-axis represents the classes of distribution for the 120 C. arabica wild accessions (blue), 11 C. arabica cultivars (red) and BA-10 genotype evaluated. The Y-axis shows the count of C. arabica genotypes in each category. [file DataSheet_1.zip › Supplementary Figure 4.JPEG]

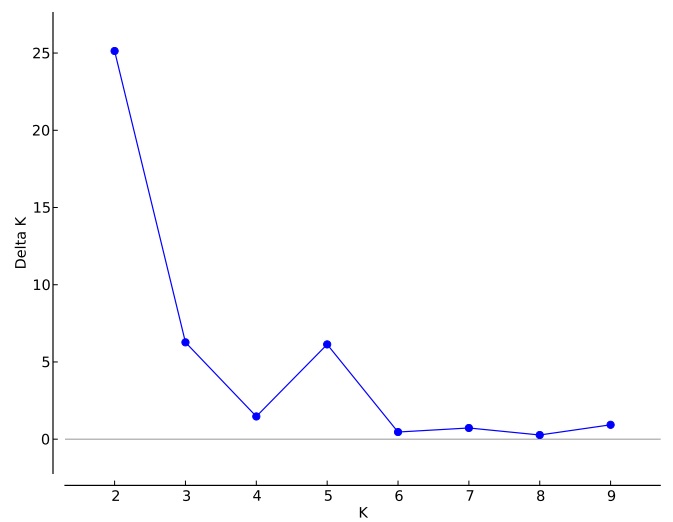

Supplement: Supplementary Figure 1 — Histogram of the disease distribution, values of response to Bacterial Halo Blight obtained in field evaluation (Mohan et al., 1978; Ito et al., 2008). The X-axis represents the classes of distribution for the 120 C. arabica wild accessions (blue), 11 C. arabica cultivars (red) and BA-10 genotype evaluated. The Y-axis shows the count of C. arabica genotypes in each category. [file DataSheet_1.zip › Supplementary Figure 5.JPEG]

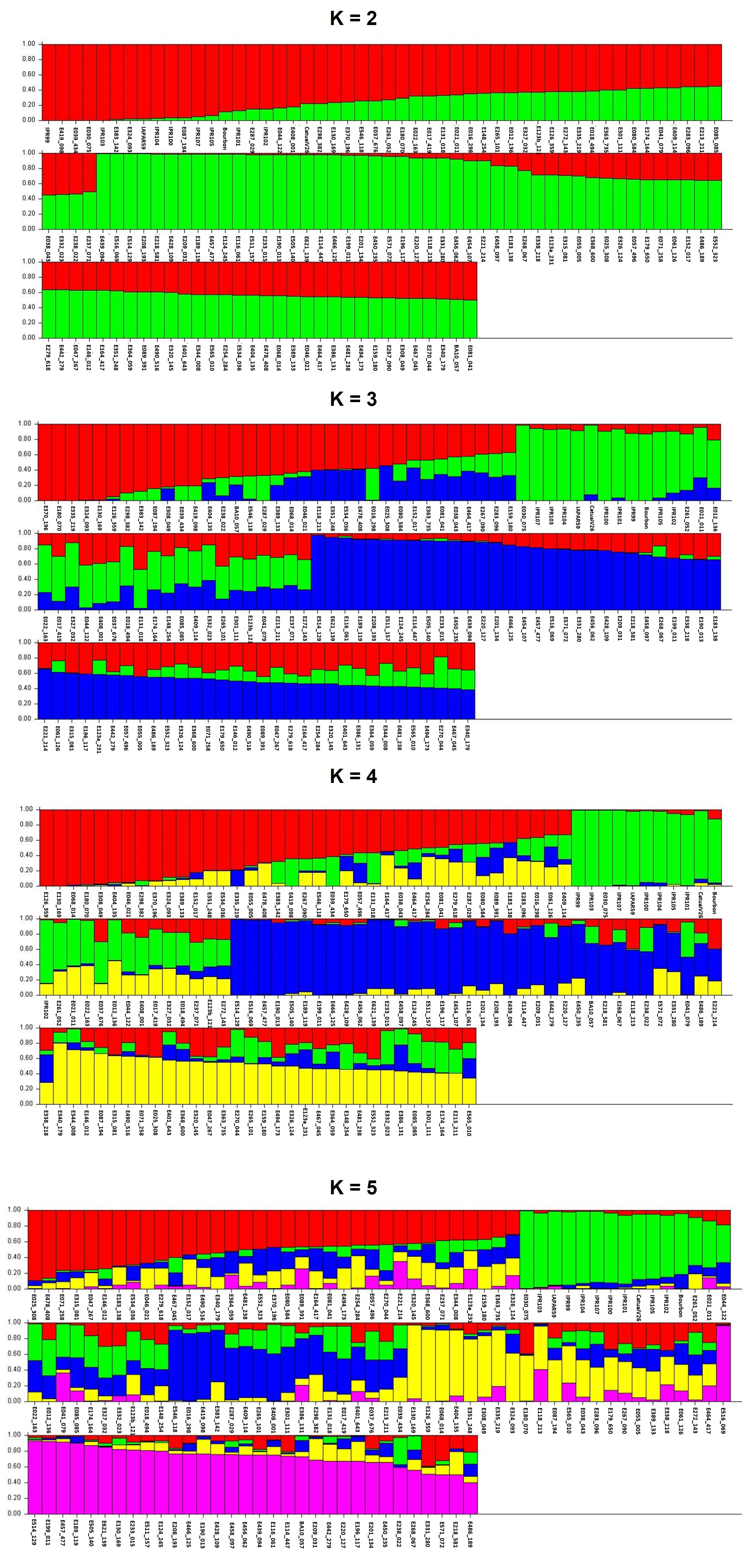

Supplement: Supplementary Figure 1 — Histogram of the disease distribution, values of response to Bacterial Halo Blight obtained in field evaluation (Mohan et al., 1978; Ito et al., 2008). The X-axis represents the classes of distribution for the 120 C. arabica wild accessions (blue), 11 C. arabica cultivars (red) and BA-10 genotype evaluated. The Y-axis shows the count of C. arabica genotypes in each category. [file DataSheet_1.zip › Supplementary Figure 6.JPEG]

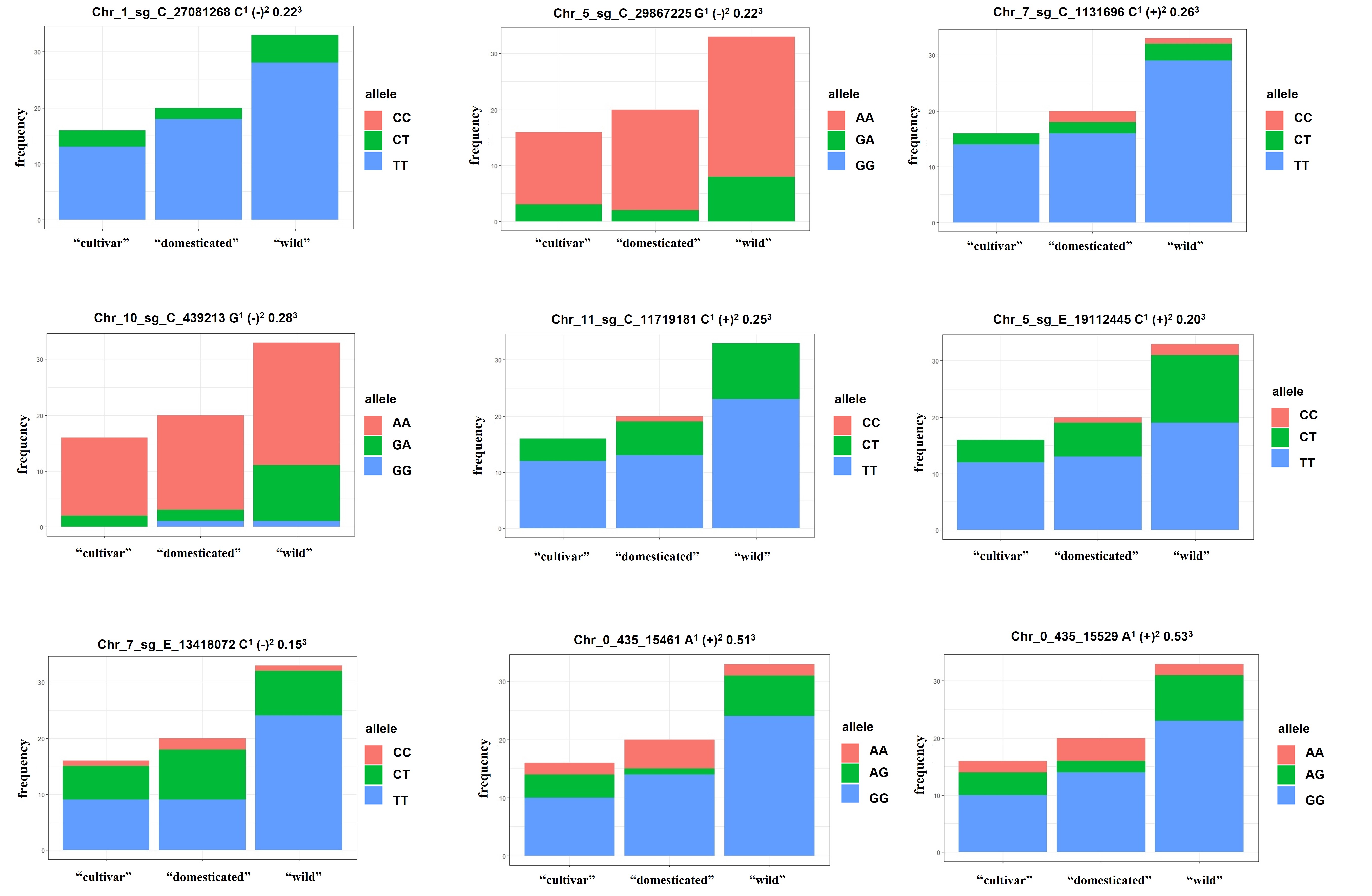

Supplement: Supplementary Figure 1 — Histogram of the disease distribution, values of response to Bacterial Halo Blight obtained in field evaluation (Mohan et al., 1978; Ito et al., 2008). The X-axis represents the classes of distribution for the 120 C. arabica wild accessions (blue), 11 C. arabica cultivars (red) and BA-10 genotype evaluated. The Y-axis shows the count of C. arabica genotypes in each category. [file DataSheet_1.zip › Supplementary Figure 7.JPEG]

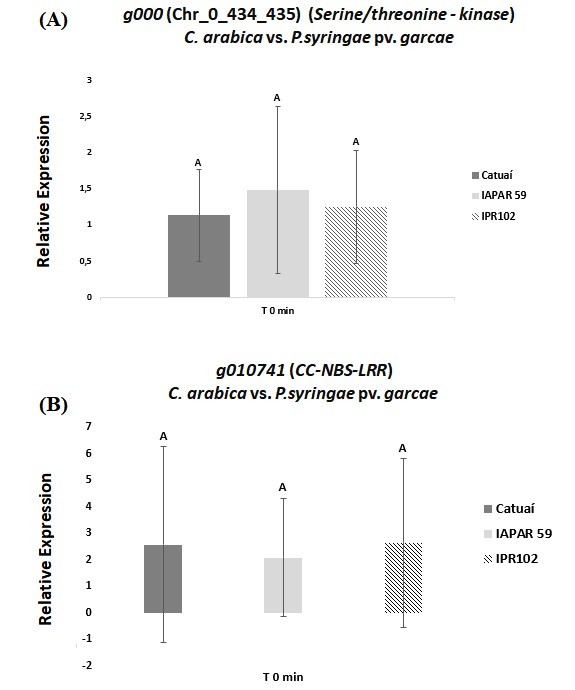

Supplement: Supplementary Figure 1 — Histogram of the disease distribution, values of response to Bacterial Halo Blight obtained in field evaluation (Mohan et al., 1978; Ito et al., 2008). The X-axis represents the classes of distribution for the 120 C. arabica wild accessions (blue), 11 C. arabica cultivars (red) and BA-10 genotype evaluated. The Y-axis shows the count of C. arabica genotypes in each category. [file DataSheet_1.zip › Supplementary Figure 8.JPEG]

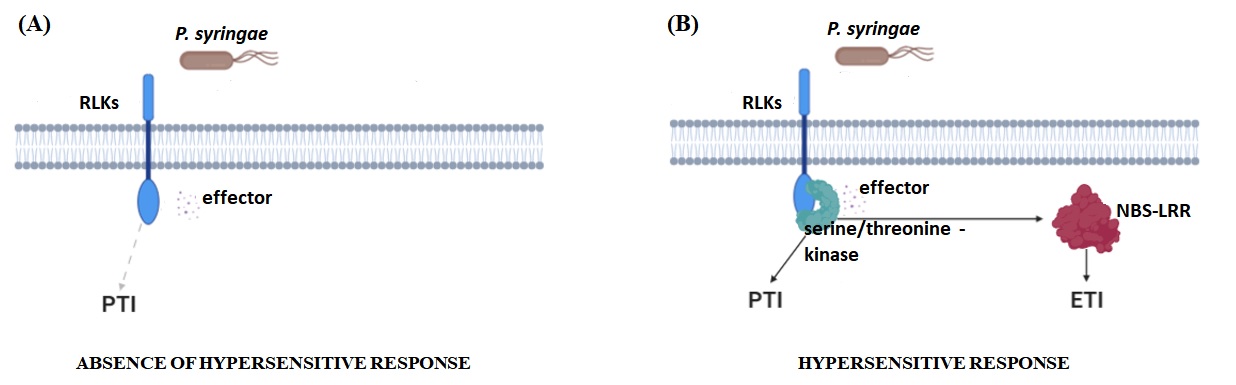

Supplement: Supplementary Figure 1 — Histogram of the disease distribution, values of response to Bacterial Halo Blight obtained in field evaluation (Mohan et al., 1978; Ito et al., 2008). The X-axis represents the classes of distribution for the 120 C. arabica wild accessions (blue), 11 C. arabica cultivars (red) and BA-10 genotype evaluated. The Y-axis shows the count of C. arabica genotypes in each category. [file DataSheet_1.zip › Supplementary Figure 9.JPEG]
